# The Potential Impact of HNRNPA2B1 on Human Cancers Prognosis and Immune Microenvironment

**DOI:** 10.1155/2024/5515307

**Published:** 2024-09-05

**Authors:** Tao Huang, Gang Zhu, Fan Chen

**Affiliations:** Department of Neurosurgery Tangdu Hospital Fourth Military Medical University, Xi'an, China

## Abstract

HNRNPA2B1 is a member of the HNRNP family, which is associated with telomere function, mRNA translation, and splicing, and plays an important role in tumor development. To date, there have been no pan-cancer studies of HNRNPA2B1, particularly within the TME. Therefore, we conducted a pan-cancer analysis of HNRNPA2B1 using TCGA data. Based on datasets from TCGA, TARGET, Genotype-Tissue Expression, and Human Protein Atlas, we employed a range of bioinformatics approaches to explore the potential oncogenic role of HNRNPA2B1. This included analyzing the association of HNRNPA2B1 expression with prognosis, tumor mutation burden (TMB), microsatellite instability (MSI), immune response, and immune cell infiltration of individual tumors. We further validated the bioinformatic findings using immunohistochemistry techniques. HNRNPA2B1 was found to be differentially expressed across most tumor types in TCGA's pan-cancer database and was predictive of poorer clinical staging and survival status. HNRNPA2B1 expression was also closely linked to TMB, MSI, tumor stemness, and chemotherapy response. HNRNPA2B1 plays a significant role in the TME and is involved in the regulation of novel immunotherapies. Its expression is significantly associated with the infiltration of macrophages, dendritic cells, NK cells, and T cells. Furthermore, HNRNPA2B1 is closely associated with immune checkpoints, immune-stimulatory genes, immune-inhibitory genes, MHC genes, chemokines, and chemokine receptors. We performed a comprehensive evaluation of HNRNPA2B1, revealing its potential role as a prognostic indicator for patients and its immunomodulatory functions.

## 1. Introduction

Cancer is the leading cause of death worldwide [1]. Despite extensive research, a comprehensive understanding of its development mechanisms and effective treatments remains elusive [1, 2]. The complexity of cancer is further compounded by the presence of multiple genetic alterations within cancer cells, which result in the expression of a diverse array of antigens on the cell surface [3]. Over recent years, immunotherapy against cancer cell surface antigens has emerged as a promising cancer treatment, especially immune checkpoint blockade therapy; however, it has been found in clinical applications that cancer cells also display complex immunotherapy-resistant properties that affect the effectiveness of immunotherapy [4]. So far, multiple immune escape mechanisms have been reported in cancer cells, among which N6-methyladenine (m6A) has been reported to affect the immune microenvironment and mediate immune escape in a variety of cancers [5, 6, 7, 8]. m6A plays an important role in RNA nucleation, RNA–protein interactions, mRNA stability, mRNA splicing, mRNA metabolism, and mRNA translation [9]. Dysregulation of m6A expression has been implicated in the promotion of tumorigenesis, exerting its effects through modulation of oncogene expression and alteration of immune cell infiltration, among other mechanisms [10].

HNRNPA2B1 is a member of the hnRNP family of RNA-binding proteins, and m6A-modified nuclear readers which can bind to RGm6AC-containing sites on nuclear RNAs [11]. HNRNPA2B1 is associated with pre-mRNAs in the nucleus and plays a key role in primary microRNA processing, selective splicing, mRNA metabolism, as well as transport [12]. Accumulating evidence from previous studies indicates that tumor neoantigens can modulate the immune cell infiltration within the tumor microenvironment (TME) and elicit antitumor immune responses, altering the response to tumor immunotherapy [8, 9]. HNRNPA2B1 has been shown to exert a pivotal influence on tumor development, formation of TME, antigen-specific antitumor immunity, and maintenance of tumor stemness [13, 14, 15, 16, 17]. Jiang et al. [13] documented that HNRNPA2B1 is overexpressed in multiple myeloma and can promote the proliferation of myeloma cells. Zhao et al. [15] elucidated that HNRNPA2B1 facilitates the encapsulation of miR-934 into exosomes, downregulate PTEN expression, and activate PI3K/AKT signaling pathway to induce M2 macrophage polarization. Yuan et al. [17] reported that HNRNPA2B1 is extensively expressed within the glioma microenvironment and can promote the maintenance of stemness in glioma cells. Kong et al. [18] have identified a significant correlation between HNRNPA2B1 and the efficacy of novel immunotherapies in urothelial carcinoma. They have further developed a nomogram predicated on HNRNPA2B1 expression levels to provide personalized predictions of response to atezolizumab, a PD-L1 inhibitor. These findings collectively suggest that HNRNPA2B1 may serve as a promising biomarker for the prognosis of cancer and the individualized assessment of responses to innovative immunotherapies.

However, the role of HNRNPA2B1 in human pan-cancer development, immune microenvironment, immunotherapy, and prognosis has not been systematically analyzed so far. In our study, we analyzed the expression of HNRNPA2B1 in various types of cancer and its relationship with patient prognosis. Furthermore, we delved into the interplay between HNRNPA2B1 expression and tumor immunity, as well as its relevance to immunotherapy, including immune cell infiltration, immune-related genes, immune checkpoints, chemokines and their receptors, immunotherapeutic response, and novel immunotherapies. We also explored the association of HNRNPA2B1 expression with other RNA modification patterns as well as tumor stemness. Our results provide a comprehensive insight into the role of HNRNPA2B1 in pan-cancer, focusing on its impact on the TME and its potential in anticancer immunotherapy, and provide a basis for developing new immunotherapy strategies.

## 2. Results

### 2.1. Expression Landscape of HNRNPA2B1 in Pan-Cancer

**Figure 1:**
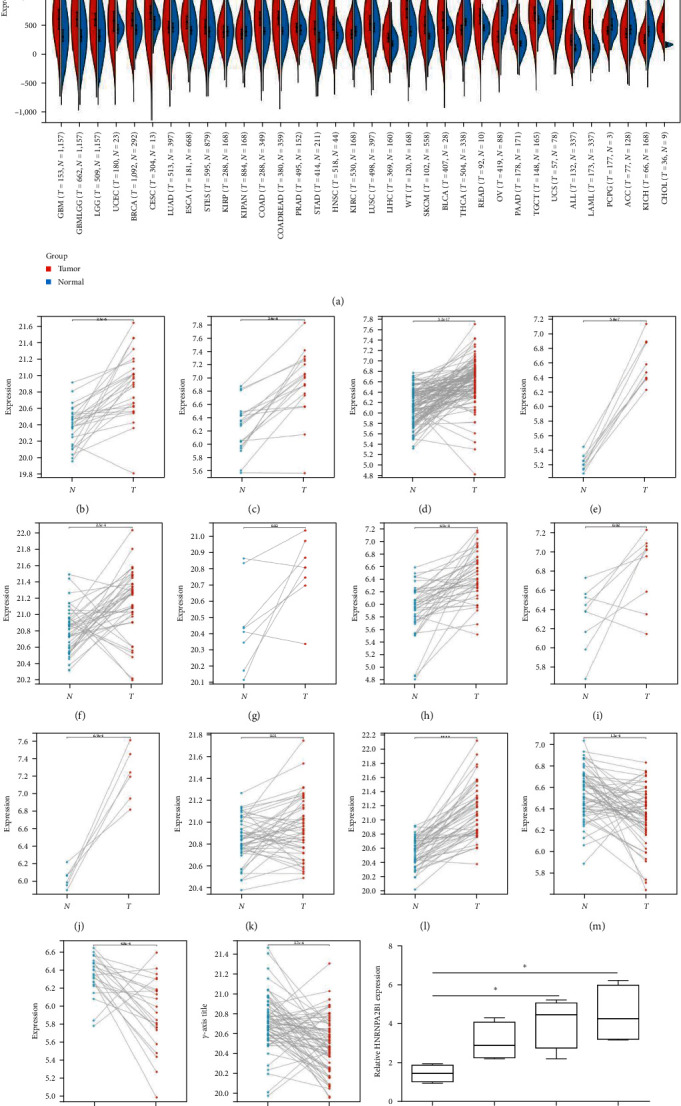
Pan-cancer HNRNPA2B1 expression: (a) pan-cancer expression of HNRNPA2B1 between tumor tissues and normal tissues. Pan-cancer paired HNRNPA2B1 expression. (b–o) Pan-cancer differential expression of HNRNPA2B1 in paired tumor and adjacent normal tissues in indicated tumor types from TCGA and TARGET database, (b) TCGA-STAD; (c) TCGA-BLCA; (d) TCGA-BRCA; (e) TCGA-CHOL; (f) TCGA-COAD; (g) TCGA-ESCA; (h) TCGA-HNSC; (i) TCGA-READ; (j) TARGET-WT; (k) TCGA-LIHC; (l) TCGA-LUSC; (m) TCGA-THCA; (n) TCGA-KICH; and (o) TCGA-KIRC. (p) Relative qPCR expression levels of HNRNPA2B1 in HA1800, HS683, U251, and A172 cells.  ^*∗*^*p* < 0.05;  ^*∗∗*^*p* < 0.01;  ^*∗∗∗*^*p* < 0.001; and  ^*∗∗∗∗*^*p* < 0.0001; –, not significant.

We calculated the difference in expression between normal and tumor samples in each tumor and performed differential significance analysis using unpaired Wilcoxon rank-sum and signed rank tests, we observed significant upregulation in 24 tumors (Figure 1(a)), such as GBM, GBMLGG, LGG, UCEC, BRCA, CESC, ESCA, STES, COAD, COADREAD, PRAD, STAD, HNSC, LUSC, LIHC, WT, SKCM, BLCA, READ, PAAD, TGCT, ALL, LAML, and CHOL. Meanwhile, we observed significant low HNRNPA2B1 expression in seven tumors, such as KIPAN, KIRC, THCA, OV, UCS, ACC, and KICH.

We further analyzed matched tumor and paraneoplastic tissues in the TCGA pan-cancer cohort and HNRNPA2B1 was significantly highly expressed in STAD, BLCA, BRCA, CHOL, COAD, ESCA, HNSC, READ, WT, LIHC, and LUSC (Figures 1(b), 1(c), 1(d), 1(e), 1(f), 1(g), 1(h), 1(i), 1(j), 1(k), and 1(l)), while significantly low in THCA, KICH, and KIRC (Figures 1(m), 1(n), and 1(o)), consistent with the above results in both normal and tumor samples. Meanwhile, we further confirmed the expression of HNRNPA2B1 in different grades of glioma cells using qRT-PCR. HNRNPA2B1 expression was significantly higher in HS683, U251, and A172 cells than in HA-1800 cells, and HNRNPA2B1 expression was greater in U251 and A172 cells than in HS683 cells (Figure 1(p)).

**Figure 2:**
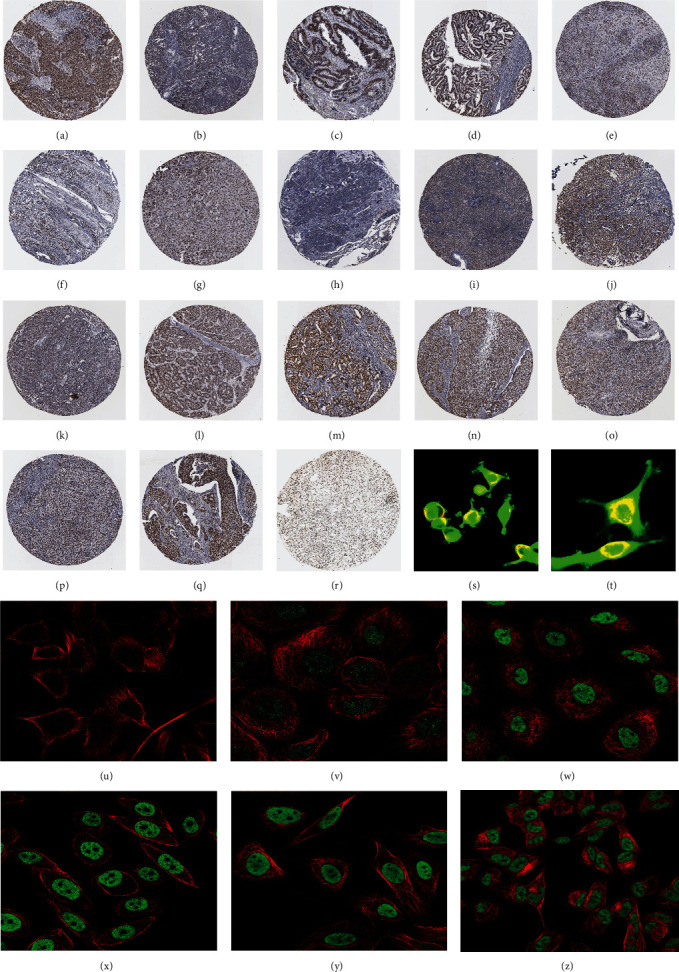
The expression of HNRNPA2B1 was detected by immunohistochemistry and the subcellular location of HNRNPA2B1 was detected by immunofluorescence. Immunohistochemical results from HPA database: (a) breast cancer; (b) cervical cancer; (c) colorectal cancer; (d) endometrial cancer; (e) GBM; (f) head and neck cancer; (g) liver cancer; (h) lung cancer; (i) lymphoma; (j) melanoma; (k) ovarian cancer; (l) pancreatic cancer; (m) prostate cancer; (n) skin cancer; (o) stomach cancer; (p) testis cancer; (q) urothelial cancer; (r) KIRC; immunofluorescence results: (s) GL261, (t) MDA-MB-231; immunofluorescence results from HPA database: (u) U251-MG; (v) A-431; (w) A549; (x) PC-3; (y) U-2 OS; and (z) SH-SY5Y.

We also employed IHC assay and immunofluorescence techniques to assess the expression of HNRNPA2B1 in tumor tissues and its localization in tumor cells. First, we examined the available data from the HPA database. The IHC results showed that HNRNPA2B1 was highly expressed in breast cancer, cervical cancer, colorectal cancer, endometrial cancer, GBM, head and neck cancer, liver cancer, lung cancer, lymphoma, melanoma, ovarian cancer, pancreatic cancer, prostate cancer, skin cancer, stomach cancer, testis cancer, urothelial cancer, KIRC, etc. (Figures 2(a), 2(b), 2(c), 2(d), 2(e), 2(f), 2(g), 2(h), 2(i), 2(j), 2(k), 2(l), 2(m), 2(n), 2(o), 2(p), 2(q), and 2(r)). We further clarified the subcellular localization of HNRNPA2B1 by immunofluorescence assay using glioma GL261 cells and breast cancer MDA-MB-231 cells, which showed that HNRNPA2B1 was expressed predominantly in the nucleus and to a lesser extent in the cytoplasm (Figures 2(s) and 2(t)). In addition, the immunofluorescence results from HPA database showed that HNPA2B1 was mainly expressed in the nucleus of U251-MG, A-431, A549, PC-3, U-2, and SH-SY5Y cell line, and there were also some expressions in the cytoplasm (Figure 2(u), 2(v), 2(w), 2(x), 2(y), and 2(z)). We next examined and compared the expression of HNRNPA2B1 in matched cancer and paraneoplastic tissue samples from our hospital, and higher expression levels of HNRNPA2B1 were also observed in tumor tissues from ESCA, STAD, COAD, READ, LIHC, LUSC, BRCA, CESC, LGG, BLCA, DLBC, HNSC, GBM, PRAD, and PAAD patients, and lower expression levels of HNRNPA2B1 were observed in tumor tissues from KIRC patients (*Supplementary figure 1*).

### 2.2. Pan-Cancer Analysis of the Correlation between HNRNPA2B1 Expression and Clinical Staging

To analyze the relationship between HNRNPA2B1 and cancer progression, we calculated HNRNPA2B1 expression in different WHO cancer clinical stages in each cancer, we observed significant differences in four tumors (*Supplementary figure 1*), such as LUSC, LIHC, TGCT, and ACC. At the same time, we also found some differences between clinical stages in CESC, OV, KIPAN, and LUAD (*Supplementary figure 1*).

### 2.3. Pan-Cancer Analysis of the Monitoring Value of HNRNPA2B1 on Cancer Recurrence and Prognosis

We also evaluated the relationship between HNRNPA2B1 expression and patient prognosis in the pan-cancer dataset. Survival metrics for performing pan-cancer analysis included overall survival (OS), disease free survival (DFS), progress free survival (PFS), and disease-specific survival (DSS). We used Cox regression analysis to analyze the prognostic relationship between HNRNPA2B1 expression and all types of cancer, and statistical tests using logrank test to get prognostic significance. Finally, OS analysis observed that high expression of HNRNPA2B1 in nine cancers resulted in poor prognosis, including LGG, TARGET-LAML, LUAD, SARC, LIHC, LAML, ACC, KICH, GBMLGG, and low expression of HNRNPA2B1 in OV resulted in poor prognosis (*Supplementary figure 1*, *Supplementary table 2*). DSS analysis observed that high expression of HNRNPA2B1 in nine cancers resulted in poor prognosis, including GBMLGG, LGG, LUAD, LUSC, LIHC, ACC, KICH, and low expression of HNRNPA2B1 in OV with poor prognosis (*Supplementary figure 1*, *Supplementary table 3*). DFS analysis observed that high expression of HNRNPA2B1 in three cancers resulted in cancer progression, including CESC, LIHC, ACC, and low expression of HNRNPA2B1 in OV with cancer progression (*Supplementary figure 1*, *Supplementary table 4*). PFS analysis observed that high expression of HNRNPA2B1 in 10 cancers resulted in cancer progression, including GBMLGG, LGG, CESC, LUAD, PRAD, LUSC, LIHC, UVM, ACC, KICH, and low expression of HNRNPA2B1 in OV with poor (*Supplementary figure 1*, *Supplementary table 4*).

**Figure 3:**
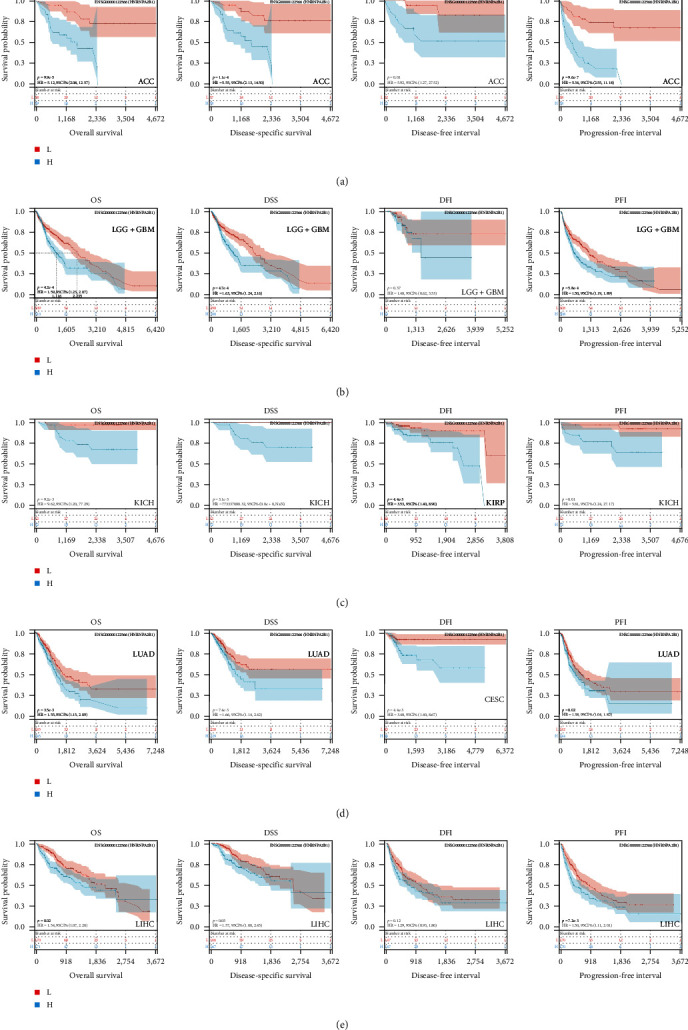
Kaplan–Meier survival of HNRNPA2B1 expression: (a–e) pan-cancer Kaplan–Meier OS, DSS, DFI, and PFI of HNRNPA2B1 in indicated tumor types from TCGA database. (a) ACC; (b) LGG + BGM; (c) KICH + KIRP; (d) LUAD + CESC; and (e) LIHC. The median value of HNRNPA2B1 in each tumor was taken as the cut-off value.

We further analyzed OS, DSS, DFS, and PFS using Kaplan–Meier analysis (Figure 3, *Supplementary figure 1*). Kaplan–Meier OS analysis showed that high expression of HNRNPA2B1 was a risk factor for patients with ACC, GBMLGG, KICH, LUAD, LIHC, LAML, TARGET-LAML, LGG, PRAD, and SARC, and a favorable factor for patients with STAD and UCEC. Kaplan–Meier DSS analysis showed that high expression of HNRNPA2B1 was a risk factor for patients with ACC, GBMLGG, KICH, LUAD, LIHC, LGG, PRAD, and SARC. Kaplan–Meier DFS analysis showed that high expression of HNRNPA2B1 was a risk factor for the progression of ACC, GBMLGG, KIRP, CESC, LIHC, COAD, and KIPAN, as well as a favorable factor for resistance to OV progression. Kaplan–Meier PFS analysis showed that high expression of HNRNPA2B1 was a risk factor for the progression of ACC, GBMLGG, LUAD, LGG, CESC, LIHC, PRAD, and UVM. Based on the comprehensive analysis of Cox regression analysis and Kaplan Meier analysis on OS, DSS, DFS, and PFS, we found that the high expression of HNRPA2B1 is an unfavorable prognostic factor for LGG, GBMLGG, ACC, LIHC, KICH, LAML, CESC, LUAD, SARC, and PRAD, while the high expression of HNRPA2B1 is a favorable prognostic factor for OV.

### 2.4. Pan-Cancer Analysis of HNRNPA2B1-Associated Genetic Alterations and RNA Modifications

**Figure 4:**
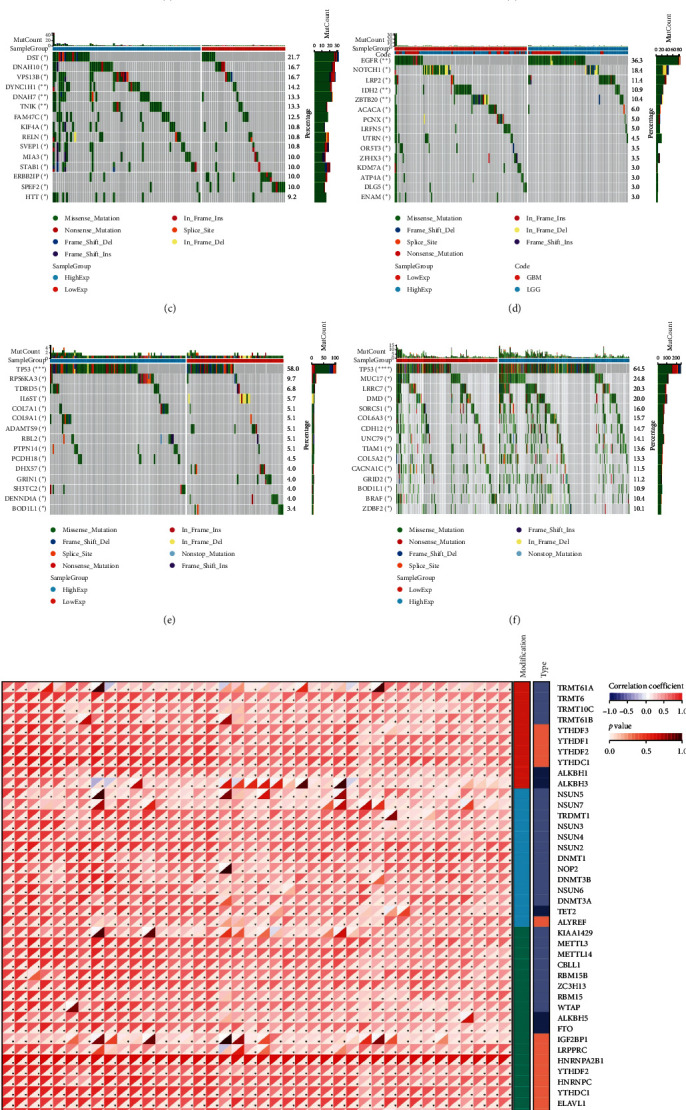
Relationship between HNRNPA2B1 expression and mutation landscape, RNA modifications: (a) STAD; (b) COAD; (c) CESC; (d) LGG + GBM; (e) LIHC; (f) LUAD; and (g) RNA modification. The median value of HNRNPA2B1 in each tumor was taken as the cut-off value.  ^*∗*^*p* < 0.05;  ^*∗∗*^*p* < 0.01; and  ^*∗∗∗*^*p* < 0.001.

In general, changes in genetics and epigenetics lead to modifications in gene expression [19]. We divided the cancer samples into high- and low-expression groups according to the expression value of HNRNPA2B1 and further analyzed the differences in somatic mutation distribution between the two groups. The results showed significant differences in genetic alterations between the two groups in STAD, COAD, CESC, GBMLGG, LIHC, LUAD, LGG, OV, PRAD, KIRP, and LAML patients, and Figures 4(a), 4(b), 4(c), 4(d), 4(e), and 4(f) and *Supplementary figure 1* demonstrate the TOP15 gene mutations. Especially in some classical cancer-related genes, such as TTN and TP53 in STAD (Figure 4(a)), APC and TP53 in COAD (Figure 4(b)), DST and HTT in CESC (Figure 4(c)), EGFR and IDH in GBMLGG (Figure 4(d)), TP53 and IL6ST in LIHC (Figure 4(e)), TP53 and BEAF in LUAD (Figure 4(f)), EGFR and IDH2 in LGG (*Supplementary figure 1*), APC and RB1 in OV (*Supplementary figure 1*), TP53 and TMPRSS2 in PRAD (*Supplementary figure 1*), NOTCH2 and CUBN in KIRP (*Supplementary figure 1*), and RUNX1 and MUC16 in LAML (*Supplementary figure 1*).

We calculated the difference of HNRNPA2B1 expression across samples representing different clinical stages for each type of tumor, and used unpaired Wilcoxon rank-sum and signed rank tests to analyze the significance of the difference, using Kruskal Test to test the difference of multiple groups of samples, we observed significant differences in seven cancers, such as BRCA, ESCA, STES, SARC, STAD, PRAD, and OV (*Supplementary figure 1*).

There is growing evidence that RNA modification pathways are also dysregulated in human cancers, including m1A, m5C, and m6A, which may be ideal targets for cancer therapy [7, 8, 20]. HNRNPA2B1 is a reader for m6A modification, and we further investigated the relationship of HNRNPA2B1 with genes related to m1A, m5C, and m6A modifications. The results showed that HNRNPA2B1 correlated with m1A, m5C, and m6A modified related genes in various cancers, especially in ACC, OV, UVM, KICH, SKCM, GBM, WT, DLBC, THYM, THCA, UCEC, PCPG, and KIRP (Figure 4(g)).

### 2.5. Pan-Cancer Analysis of the Correlation between HNRNPA2B1 Expression and Immune Cell Infiltration as well as Immune Checkpoints

**Figure 5:**
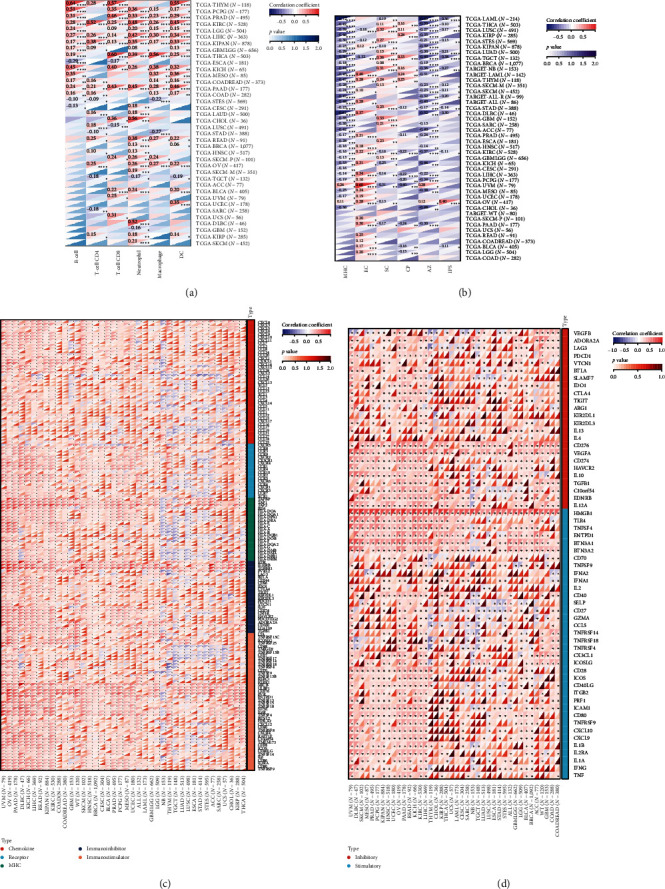
Analysis of the relationship between HNRNPA2B1 expression and tumor microenvironment: (a) the correlation between HNRNPA2B1 and infiltration level of IMMUNE cells using TIMER database; (b) the correlation between HNRNPA2B1 and IPS; (c) the correlation between HNRNPA2B1 and immune regulatory genes; and (d) the correlation between HNRNPA2B1 and immune checkpoint genes.  ^*∗*^*p* < 0.05;  ^*∗∗*^*p* < 0.01; and  ^*∗∗∗*^*p* < 0.001.

To analyze the relationship between HNRNPA2B1 expression and immune cell infiltration in the tumor immune microenvironment, we evaluated the infiltration scores of B cells, CD4+ T cells, CD8+ T cells, neutrophil, macrophage, and DC in each patient in each tumor according to the expression of HNRNPA2B1 using TIMER analysis, and finally we observed that the expression of this gene was significantly related to the immune infiltration in 37 cancer species (Figure 5(a)). In most cancers, the expression of HNRNPA2B1 showed a positive correlation with immune cell infiltration (Figure 5(a)). We also assessed major histocompatibility complex molecular (MHC), effector cells, immunosuppressive cells, immune checkpoints, AZ, and immunophenoscore (IPS) infiltration scores for each patient in each tumor based on HNRNPA2B1 expression using IPS analysis, and ultimately we observed that HNRNPA2B1 expression was significantly associated with immune infiltration in almost all cancer types (Figure 5(b)). We further used xCELL analysis to evaluate the aDC, adipocytes, astrocytes, B-cells, basophils, macrophages, Macrophages_M1, Macrophages_M2, Mast_cells, ImmuneScore, StromaScore, MicroenvironmentScore, and other 67 immune-related features of infiltration scores for every patient in each tumor, we found that the expression of HNRNPA2B1 correlated with immune infiltration in all cancers (*Supplementary figure 1*). After applying the CIBERSOR method to assess the immune cell infiltration in the TME across pan-cancers, we observed a consistent result: The expression of HNRNPA2B1 is correlated with immune infiltration in all types of cancer (*Supplementary figure 1*).

To analyze the correlation between the expression of HNRNPA2B1 and immune pathways, we collected the expression data of 150 marker genes across five immune pathway types (chemokine, receptor, MHC, Immunoinhibitor, Immunostimulator) [8, 21] in each sample by reviewing the literature. We then calculated the Pearson correlation coefficients between HNRNPA2B1 expression and these marker genes. Our findings indicate that HNRNPA2B1 is correlated with most immunomodulators across pan-cancers, as illustrated in Figure 5(c). Additionally, after conducting an analysis of immune checkpoint-associated genes [22], we got similar results (Figure 5(d)).

### 2.6. Pan-Cancer Analysis of HNRNPA2B1 Expression in Correlation with the Immune Score, TMB, MSI, as well as Stemness

**Figure 6:**
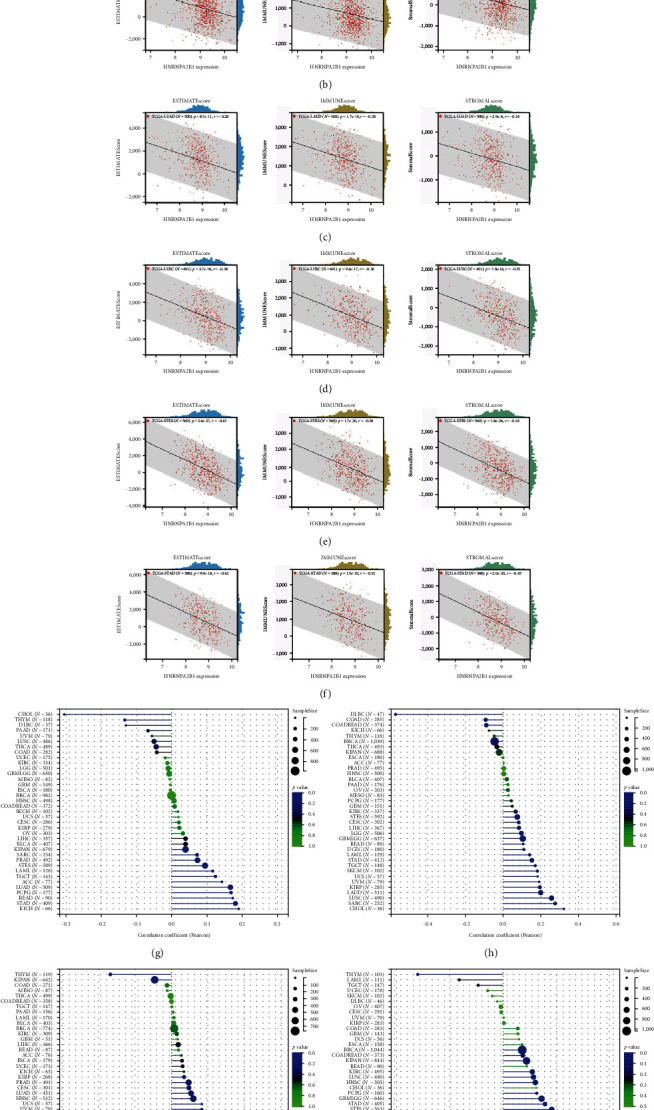
Correlation analysis between ESTIMATE scores and HNRNPA2B1 expression. The correlation between ESTIMATEscore, immunescore, stromalscore, and HNRNPA2B1: (a) ACC; (b) BRCA; (c) LUAD; (d) LUSC; (e) STES; and (f) STAD. The correlation between immunetherapy, tumor stemness, and HNRNPA2B1: (g) TMB; (h) MSI; (i) Stemness; and (j) HRD.

To further validate the correlation between HNRNPA2B1 expression and immune infiltration, we calculated the ESTIMATEScore, ImmuneScore, and StromalScore for each cancer sample, respectively. The results indicate that HNRNPA2B1 expression is significantly associated with these scores in the majority of cancers. In ACC, BRCA, LUAD, LUSC, STES, and STAD, high expression of HNRNPA2B1 showed a negative correlation with ESTIMATEScore, ImmuneScore and StromalScore, suggesting that the high expression HNRNPA2B1 group had a low degree of immune cell infiltration, which may be insensitive to immune checkpoint treatment (Figures 6(a), 6(b), 6(c), 6(d), 6(e), and 6(f)). In other cancer types, such as ESCA, GBM, KIRP, LAML, SARC, SKCM, TGCT, etc., HNRNPA2B1 expression also showed a negative correlation with immune infiltration (*Supplementary figure 1*).

The potential association between HNRNPA2B1 expression and the immunotherapeutic response has been demonstrated. Building on this, we further analyzed the potential applications of HNRNPA2B1 expression levels across different cancers. Studies have reported that the major clinically validated biomarkers reflecting the response to checkpoint blockade immunotherapy include TMB and MSI [8, 23]. We integrated TMB and HNRNPA2B1 gene expression data from pan-cancer samples and observed a significant association between HNRNPA2B1 and TMB in multiple tumors. Specifically, we found significant positive associations in four tumor types: LUAD, STES, STAD, and PCPG (Figure 6(g)). We used the same method to analyze the association between MSI and HNRNPA2B1 gene expression in pan-cancer samples, and we found that the expression levels of HNRNPA2B1 were significantly correlated with MSI in several tumors, with significant positive correlation in eight tumors: GBMLGG, LUAD, LGG, SARC, KIRP, STAD, LUSC, and TGCT, and in one tumor was significantly negatively correlated: DLBC (Figure 6(h)). We also calculated tumor stemness scores of pan-cancer samples by methylation characteristics, integrating the stemness index, and HNRNPA2B1 gene expression data of the samples, we observed that HNRNPA2B1 expression was significantly correlated with tumor stemness index in 13 tumors, including a significant positive correlation in 11 tumors: GBMLGG, LUAD, LGG, STES, SARC, STAD, PRAD, HNSC, LUSC, PCPG, and SKCM, and significant negative correlation in two tumors: KIPAN, THYM (Figure 6(i)). Homologous recombination deficiency (HRD) produces specific, quantifiable, and stable genomic alterations, and HRD status is a key indicator of treatment choice and prognosis for a variety of tumors [22]. Clinical findings confirm that HRD status is highly correlated with sensitivity to platinum-based chemotherapeutic agents and PARP inhibitors. By analyzing the expression of HRD and HNRNPA2B1 in pan-cancer samples, we found that they were significantly correlated in 17 tumors, with significant positive correlation in 16 tumors, such as GBMLGG, LUAD, LGG, BRCA, STES, SARC, KIPAN, STAD, PRAD, HNSC, KIRC, LUSC, LIHC PAAD, BLCA, and ACC, and significantly negatively correlated in one tumor: THYM (Figure 6(j)).

### 2.7. Functional Enrichment Analysis of HNRNPA2B1 Expression in Pan-Cancer

**Figure 7:**
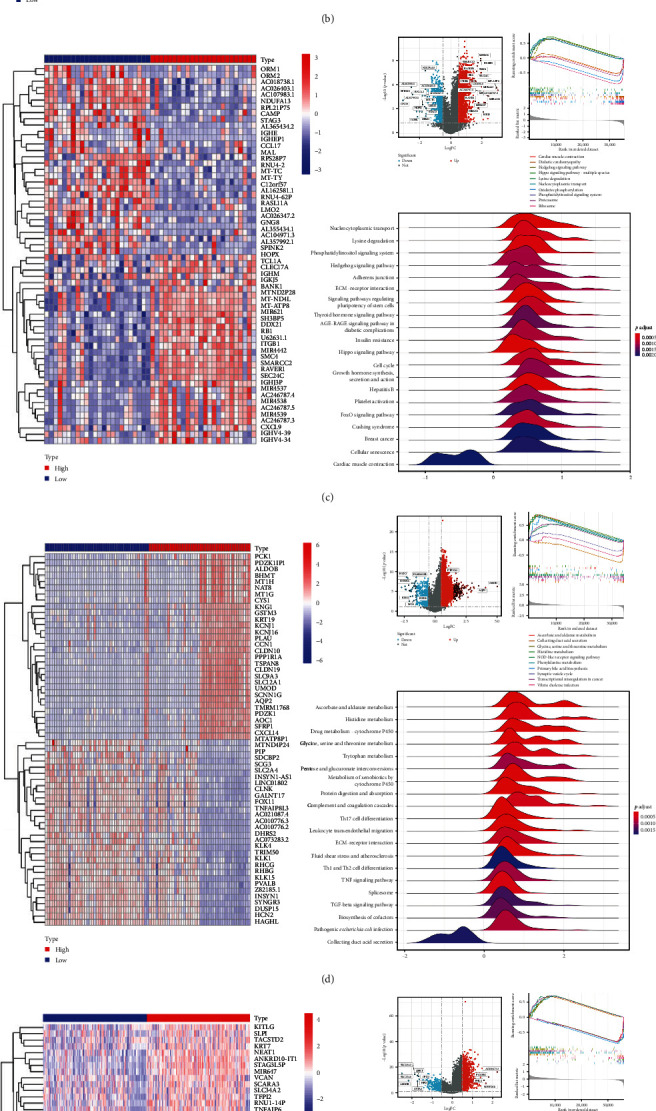
GSEA of HNRNPA2B1 in pan-cancer: (a) BLCA; (b) CHOL; (c) DLBC; (d) KICH; (e) KIRP; and (f) READ.

To assess the impact of HNRNPA2B1 expression on cancer cell function, we evaluated the pathways by which HNRNPA2B1 may be involved in the use of gene set enrichment analysis (GSEA) in pan-cancer. The results showed that in BRCA, HNSC, LIHC, MESO, PCPG, and SKCM, HNRNPA2B1 expression was significantly associated with multiple pathways, such as immune-related pathways, DNA replication, mismatch repair, and cell cycle (Figures 7(a), 7(b), 7(c), 7(d), 7(e), and 7(f)). In addition to the above tumors, we also found that HNRNPA2B1 expression in ACC, COAD, GBM, LGG, OV, STAD, LIHC, LUAD, SKCM, and UCS is closely related to spliceosomes, ribosomes, nucleocytoplasmic transport, p53-signaling pathway, RNA degradation, and surveillance pathway in addition to immune-related pathways, which indicates that HNRNPA2B1 plays an important role in both tumor development and immunotherapy (*Supplementary figure 1*).

We next constructed PPI networks for breast, colorectal, gastric, and lung cancers using the STRING database. The differentially expressed genes between normal and cancerous tissues were uploaded to the STRING website to analyze the interactions of these proteins. *Supplementary figure 1* shows the protein interactions between the Top 15 hub genes centered on HNRNPA2B1 in breast cancer (*Supplementary figure 1*), colorectal cancer (*Supplementary figure 1*), gastric cancer (*Supplementary figure 1*), and lung cancer (*Supplementary figure 1*), respectively. The analyses of GO function and KEGG pathways indicated that HNRNPA2B1 was principally involved in gene expression, DNA and mRNA metabolic process, miRNA, mRNA and RNA binding, exosome, primary miRNA processing, protein phosphorylation and modification process, and immune-related pathways (*Supplementary table 2*, *Supplementary table 3*, *Supplementary table 4*, and *Supplementary table 5*).

## 3. Discussion

In recent years, novel immunotherapies have demonstrated significant potential in cancer treatment and have emerged as one of the most critical therapeutic strategies for cancer treatment [24]. Novel immunotherapies, mainly including PD-1 and PD-L1, are the most studied in recent years immune checkpoint blockade therapies, which can induce durable anticancer responses [25]. HNRNPA2B1, acting as the “reader” of m6A modification, can influence primary microRNA processing, mRNA metabolism, and transport, thus influencing immune cell infiltration and the development of the tumor immune microenvironment [5, 15]. HNRNPA2B1 could mediate miR-934 packaging into the exosomes of colorectal cancer cells and then transfer exosomal miR-934 to macrophages, thereby inducing macrophage enrichment and M2-type polarization [15]. In glioma, HNRNPA2B1 can package circNEIL3 into exosomes and transmit them to infiltrating tumor-associated macrophages, stabilizing IGF2BP3 so that macrophages in the TME can acquire immunosuppressive properties, thereby promoting glioma cell proliferation [16]. In adult T-cell leukemia or lymphoma, splice site mutations in HNRNPA2B1 lead to intron retention and premature truncation [26]. These alterations provide a mechanism for targeted truncation of the relevant structural domains within affected genes, resulting in protein function changes. These studies suggest that HNRNPA2B1 could be a promising target for antitumor immunotherapy. In this study, we utilized a pan-cancer dataset to evaluate the role of HNRNPA2B1.

First, we determined the expression levels of HNRNPA2B1 in cancer and normal tissues in a pan-cancer database, which was further validated in matched tumor and paraneoplastic tissues. We found that HNRNPA2B1 was highly expressed in 24 tumors and lowly expressed in seven tumors. In matched samples, we found that HNRNPA2B1 was significantly highly expressed in STAD, BLCA, BRCA, CHOL, COAD, ESCA, HNSC, READ, WT, LIHC, and LUSC, while significantly low in THCA, KICH, and KIRC. Our results are similar to previous reports that HNRPA2B1 is downregulated in renal carcinoma (KICH, KIRC) at both mRNA and protein levels [27]. It has also been demonstrated that HNRNPA2B1 is a reader of m6A, which is downregulated in renal cancer, and that this downregulation is associated with a poorer prognosis [28]. This was consistent with previous studies in gastric, colon, and breast cancer [29]. Protein information of HNRNPA2B1 was explored by immunohistochemistry, and we found that protein levels of HNRNPA2B1 were highly expressed in several cancers.

Analysis of HNRNPA1B1 expression in different clinical stages of cancers confirmed that HNRNPA2B1 plays an important role in the progression of LUSC, LIHC, TGCT, ACC, CESC, OV, KIPAN, and LUAD. Kaplan–Meier and univariate Cox regression analysis for survival assessment showed that high expression of HNRNPA2B1 was a risk factor for LGG, GBMLGG, ACC, LIHC, KICH, LAML, CESC, LUAD, SARC, and PRAD patients and a protective factor for OV, and the prognostic impact of HNRNPA2B1 was consistent with expression in these cancers. HNRNPA2B1 has been reported to be highly expressed in glioma and is closely related to the poor prognosis of glioma patients [17, 30]. Mechanistically, HNRNPA2B1 induces HMGCR through the stabilization of SREBP2 mRNA, thereby triggering the cholesterol de novo synthesis and uptake, which in turn promotes glioma cell proliferation, glioma stem cell self-renewal, and tumorigenesis [31]. In addition, HNRNPA2B1 could package circNEIL3 into exosomes and deliver it to infiltrating TAMs, thus enabling them to obtain immunosuppressive properties by stabilizing IGF2BP3, which in turn contributes to the progression of gliomas [16]. HNRNPA2B1 has also been reported to be highly expressed in lung cancer and is strongly associated with poor prognosis in lung cancer patients [32, 33]. It has been demonstrated that HNRNPA2B1-mediated m6A modification of lncRNA MEG3 facilitates tumorigenesis and metastasis of nonsmall cell lung cancer cells by regulating the miR-21-5p/PTEN axis [34]. Jin et al. [35] reported that HNRNPA2B1 was highly expressed in ACC and HNRNPA2B1 resulted in poor OS and event-free survival. It has been noted that using OS as an endpoint may reduce the accuracy of the results because the data include deaths from noncancer causes, which do not reflect tumor invasion or treatment response [19]. Accordingly, we referred to other clinical trials and selected DFI and PFI, which could more effectively reflect the influence of research factors on patients [36]. Kaplan–Meier analysis and univariate Cox regression were conducted to assess the correlation between HNRNPA2B1 expression and DFI or PFI in tumor patients. The results were consistent with those for OS and DSS, suggesting that HNRNPA2B1 is a potential pan-cancer prognostic biomarker.

Genetic and epigenetic modifications result in alterations of gene expression. Past studies have demonstrated that DNA methylation, histone modifications, and RNA modifications are key regulators of numerous biological processes critical for oncogenesis [37]. Using the median expression value of HNRNPA2B1 as a cut-off value, we found significant differences in genetic alterations between high and low expression groups in several cancers, especially in some classical cancer-related genes, such as TP53, EGFR, IDH, etc. m1A, m5C, and m6A are the most well-studied RNA modification patterns that have been shown to play a critical role in the development of multiple cancers [7, 38]. By analyzing the relationship between HNRNPA1B1 and genes involved in m1A, m5C, and m6A modifications, we confirmed that HNRNPA2B1 is associated with genes related to mRNA modifications across various cancers. There is evidence to suggest that oxidative stress and inflammatory pathways play a significant role in the genesis and progression of cancer [39]. HNRNPA2B1 is posited to modulate these pathways through the regulation of miRNA, thereby influencing the intricate interplay between oxidative stress and inflammation within the tumorigenesis [40]. This provides a new direction for research on the effect of HNRNPA2B1 on cancer progression, which could alter the modification pattern of RNA in cancer by affecting RNA modification-related genes, thereby affecting cancer progression.

Infiltration of immune cells and different immune signatures in the TME play an important role in cancer progression, treatment, and recurrence [41]. Analysis of the effect of HNRNPA2B1 expression on immune cells and immune signatures in pan-cancer revealed that HNRNPA2B1 was closely associated with immune cell infiltration and immune signatures in the majority of cancers. This suggests that HNRNPA2B1 plays a role in the immune regulation of cancer. CD8+ T cells are known to be the killer cells in the T lymphocyte population, and tumor immunotherapy can restore or enhance the effector function of CD8+ T cells in the TME [42]. It has been demonstrated that CD4+ T cells not only can play a key role in the activation and memory of cytotoxic CD8+ T cells but also that intratumor CD4+ T cells have a cytotoxic program that can directly kill cancer cells [43]. In the TME, macrophages play a crucial role in cancer development, pro-inflammatory M1-like macrophages inhibit cancer progression, while anti-inflammatory M2-like macrophages promote tumor growth and invasion [44]. We confirmed the strong correlation of HNRNPA2B1 expression with CD8+ T cells, CD4+ T cells, and macrophages using multiple approaches, including the TIMER2 database, xCELL, and the CIBERSOR database.

The ESTIMATE algorithm was used to calculate ESTIMATEScore, ImmuneScore, and StromalScore to assess stromal cell and immune cell infiltration and tumor purity [45]. Our study confirmed that elevated levels of HNRNPA2B1 expression across multiple cancers were negatively correlated with ESTIMATE Score, Immune Score, and Stromal Score. This correlation suggests that tumors with high HNRNPA2B1 expression are characterized by higher tumor purity, reduced immune cell infiltration, and potentially diminished responsiveness to immune checkpoint therapies. This is consistent with the previously reported high expression of HNRNPA2B1 leading to tumor immunotherapy resistance [35, 46]. By GSEA of HNRNPA2B1, we confirmed that it is closely associated with immune-related pathways, RNA metabolism, DNA replication, cell cycle, etc. These results suggest that HNRNPA2B1 is closely related to the regulation of tumor immune microenvironment, immunotherapy, and tumor cell proliferation. This is consistent with previously reported results on the role of HNRNPA2B1 in the regulation of the immune microenvironment, immunotherapy resistance, and effects on tumor progression [15, 32, 46]. In addition, Hui et al. [47] identified eight small molecule drugs targeting the protein HNRNPA2B1 and established a lncRNA–miRNA–mRNA (HNRNPA2B1) ceRNA network, which provides a new idea for clinical immunotherapy and the development of new therapeutic agents for bladder cancer. He et al. [48] demonstrated that HNRNPA2B1 induced upregulation of PD-L1 expression by promoting the maturation of pri-miR-146b, which inhibited the infiltration of T cells into the TME and effected the antitumor activity of anti-PD-1 immunotherapy.

## 4. Limitations

We performed a comprehensive evaluation of HNRNPA2B1, revealing its potential role as a prognostic indicator for patients and its impact on the immune microenvironment. However, despite our comprehensive and systematic analysis of HNRNPA2B1, there are some limitations in this study. First, our study only contains in vitro experiments demonstrating the expression and subcellular location of HNRNPA2B1, and in vivo experiments are yet to be performed to improve the credibility of our results. Second, although we conclude that HNRNPA2B1 expression is strongly associated with immune cell infiltration and prognosis of human cancers, we lack direct evidence that HNRNPA2B1 affects prognosis through its involvement in immune infiltration. Prospective studies on the expression of HNRNPA2B1 and its role in immune infiltration of human cancers are needed next, as well as the successful development and testing of novel antitumor immunotherapeutic agents targeting HNRNPA2B1.

To put it in a nutshell, our study presents the first comprehensive analysis of the role and mechanisms of HNRNPA2B1 in pan-cancer, with a particular focus on its implications in TME formation. Our findings unveil the potential of HNRNPA2B1 as a prognostic biomarker for patients and its immune regulatory capabilities. HNRNPA2B1 is differentially expressed in most tumor types and can predict poor clinical staging and survival status. The expression of HNRNPA2B1 correlates significantly with TMB, MSI, tumor stemness, and chemotherapeutic response, and it is implicated in the modulation of novel immunotherapies and the infiltration of a diverse array of immune cells. Our research contributes further elucidate the mechanism of HNRNPA2B1 in tumor development and provides a theoretical basis for its clinical application.

## 5. Experimental Section

### 5.1. Pan-Cancer Dataset Source and Preprocessing

We downloaded the uniformly normalized pan-cancer dataset: TCGA TARGET GTEx (PANCAN, *N* = 19131, *G* = 60499) from the UCSC (https://xenabrowser.net/) database, and we screened samples from solid tissue normal, primary solid tumor, primary tumor, normal tissue, primary blood derived cancer—bone marrow, primary blood derived cancer—peripheral blood, and finally we excluded those cancer species with less than three samples in a single cancer species, and finally obtained data for 34 cancer species.

### 5.2. mRNA and Protein Level Analysis

RNA expression data of TCGA and GTEx databases were downloaded from the UCSC Xena database (https://xenabrowser.net/datapages/). Protein levels of HNRNPA2B1 in pan-cancerous and corresponding normal tissues as well as visualization of the subcellular location of HNRNPA2B1 were obtained from the Human Protein Atlas (HPA: https://www.proteinatlas.org/) database.

### 5.3. Quantitative Real-Time PCR (qRT-PCR)

We extracted total RNA from cultured tumor cells using Trizol reagent. We used a high capacity cDNA reverse transcription kit (fermentas) to synthesize the first cDNA strand. Expression was analyzed by the following on-demand gene expression assays from applied biosystems: HNRNPA2B1 and eukaryotic 18S rRNA endogenous control. The quantitative real-time PCR was conducted in a 7900 HT fast real-time PCR system. Finally, every mRNA level was normalized to 18S rRNA and used the relative quantification (2−*ΔΔ*Ct) method to calculate the fold change.

### 5.4. Immunohistochemical Staining

Paraffin-embedded individual cancer tissues were cut longitudinally into 5-*μ*m sections and kept at 60°C in the oven for 24 hr. The tissues were then deparaffinized with xylene and hydrated with an ethanol gradient (100%–70%). Followed by continuous incubation with antigen retrieval solution (Shanghai Shunbai Biotechnology Company; Shanghai, China) and 3% H_2_O_2_ for 30 min, rinsed the slides with water and incubated with primary antibody at 4°C overnight. The following day, the slides were flushed and incubated with the corresponding secondary antibodies for 30 min, then stained with 3,3′-diaminobenzidine (DAB) and hematoxylin, respectively. Then, the slides were examined and photographed using a fluorescence microscope (Olympus, Tokyo, Japan) and analyzed using Image Pro Plus 6.0 software.

### 5.5. Bioinformatic Analysis: Survival and Prognosis

We downloaded the high-quality TCGA pan-cancer prognostic dataset and TARGET pan-cancer follow-up data from UCSC and exclude the samples with a follow-up time of less than 30 days, and furthermore, we performed log2-transformation for each expression value. We also removed cancer types with a sample number of less than 10 in a single cancer type, and finally obtained the expression data of 44 cancer types and the OS, DFS, PFS, and DSS data of the corresponding samples. The relationship between HNRNPA2B1 expression in each tumor and prognosis was analyzed using the coxph function of the R package survival, and prognostic significance was analyzed by statistical tests using the logrank test.

The expression difference of HNRNPA2B1 in samples of different clinical stages in each tumor was calculated using R, the significance analysis of the difference between the two samples was performed using unpaired Student's *t*-test, and the difference test of multiple groups of samples was performed using ANOVA.

### 5.6. Analysis of Immune Cell Infiltration in the Tumor Microenvironment

The gene expression profile of each tumor was extracted from the database separately, and the expression profile was mapped to GeneSymbol, and then used the timer method [49] of R package IOBR [50] evaluated the B-cell, T-cell CD4, T-cell CD8, neutrophil, macrophage, and DC infiltration scores of each patient in each tumor according to gene expression. At the same time, xCell method and CIBERSOR method were used to evaluate the infiltration of other immune cells in each patient in each tumor according to gene expression. Finally, the R package “ggplot2,” “ggpubr,” and “ggExtra” were used to assess the correlation between the expression of HNRNPA2B1 and the level of each immune cell infiltration in cancer (*p*  < 0.05 as significant). Furthermore, we performed coexpression analysis of HNRNPA2B1 expression and immune-related genes, including genes encoding immune activation, MHC, immunosuppression, chemokines, and chemokine receptor proteins, then using the R package “limma” and “reshape2” to visualize the results.

### 5.7. Evaluation of Response to Tumor Immunotherapy

Using the ESTIMATE algorithm of the “ESTIMATE” package to calculate the ESTIMATEScore, ImmuneScore, and StromalScore for each tumor sample. The relationship between HNRNPA2B1 expression and these three scores was assessed according to the degree of immune infiltration using the R packages “ESTIMATE” and “limma.” We also calculated the correlation between TMB, MSI, stemness, homologous recombination deficiency (HRD), and the expression of HNRNPA2B1 for each tumor.

### 5.8. Biological Functions of HNRNPA2B1 Expression in Tumor Tissues

We investigated the biological functions of HNRNPA2B1 in individual tumors by GSEA and Gene Set Variation Analysis (GSVA). GSVA gene sets were obtained from the MSigDB database, GSVA scores were first generated for each tumor, and then the tumor samples were divided into two groups of high and low expression using the median number of differential genes with the R package “limma.” Functional analysis using the R package “clusterProfiler” and “enrichplot” showed the 20 pathways with the most significant correlations. Kyoto encyclopedia of genes and genomes (KEGG) gene set from GSEA. The correlation of HNRNPA2B1 expression with multiple pathways in each tumor was analyzed, and the five pathways with the most significant positive and negative correlations were shown.

### 5.9. Statistical Analyses

All data processing and statistical analyses in this study were generated by R (version 3.6.4). The chi-square test and Student's *t*-test were used to compare the differences between the two groups, and for comparisons between three or more groups, one-way ANOVA and Kruskal–Wallis tests were used as parametric and nonparametric methods, respectively. Using the coxph function of R package SURVIVAL to establish the Cox professional hazards region expression model, the relationship between the expression of HNRNPA2B1 in each tumor and prognosis was analyzed, and statistical tests were performed using logrank test to get the significance of prognosis. Survival curves for prognostic analysis were performed by the Kaplan–Meier method, and the significance of differences was also determined using the logrank test. Pearson and distance correlation analyses were performed to calculate the relationship between HNRNPA2B1 expression in cancer samples and other RNA modification-related genes, as well as the correlation coefficients between HNRNPA2B1 expression and immune cell infiltration, immune signature scores in each cancer type. Using the scale function in R to normalize multiomics data, *p*  < 0.05 was considered statistically significant.

## Data Availability

The datasets presented in this study can be found in online repositories. The names of the repository/repositories and accession number(s) can be found in the article/Supplementary Materials. The (immunohistochemical staining) data used to support the findings of this study are included within the Supplementary Materials.

## Supplementary Materials

Supplementary 1 Figure 1: immunohistochemical results from our hospital: (a) ESCA, up: tumor, down: normal; (b) STAD, up: tumor, down: normal; (c) COAD, up: tumor, down: normal; (d) READ, up: tumor, down: normal; (e) LIHC, up: tumor, down: normal; (f) LUSC, up: tumor, down: normal; (g) KIRC, up: tumor, down: normal; (h) BRCA, up: tumor, down: normal; (i) CESC, up: tumor, down: normal; (j) LGG, up: tumor, down: normal; (k) BLCA, up: tumor, down: normal; (l) DLBC, up: tumor, down: normal; (m) HNSC, up: tumor, down: normal; (n) GBM, up: tumor, down: normal; (o) PRAD, up: tumor, down: normal; (p) PAAD, up: tumor, down: normal; (q) endometrial cancer, up: tumor, down: normal; (r) melanoma, up: tumor, down: normal; and (s) ovarian cancer, up: tumor, down: normal. Figure 2: pan-cancer HNRNPA2B1 expression in different clinical stages. (a–h) Pan-cancer differential expression of HNRNPA2B1 in clinical stages in indicated tumor types from TCGA database. (a) ACC; (b) LUSC; (c) TGCT; (d) LIHC; (e) CESC; (f) OV; (g) KIPAN; and (h) LUAD. Figure 3: univariate Cox regression analysis of HNRNPA2B1. The forest plot shows the univariate Cox regression results of HNRNPA2B1 on pan-cancer survival (a–d). (a) OS; (b) DSS; (c) DFI; and (d) PFI. Figure 4: Kaplan–Meier survival of HNRNPA2B1 expression. OS: (a–g), (a) TARGET-LAML, (b) TCGA-LAML, (c) TCGA-LGG, (d) TCGA-PRAD, (e) TCGA-SARC, (f) TCGA-STAD, and (g) TCGA-UCEC; DSS: (h–j), (h) TCGA-LGG, (i) TCGA-SARC, and (j) TCGA-PRAD; DFI: (k–m), (k) TCGA-COAD, (l) TCGA-KIPAN, and (m) TCGA-OV; PFI: (n–q), (n) TCGA-LGG, (o) TCGA-CESC, (p) TCGA-PRAD, and (q) TCGA-UVM. Figure 5: association of HNRNPA2B1 expression with mutation landscape, different clinical stages. (a) LGG; (b) OV; (c) PRAD; (d) KIRP; (e) LAML; and (f) different clinical stages.  ^*∗*^*p* < 0.05;  ^*∗∗*^*p* < 0.01;  ^*∗∗∗*^*p* < 0.001. Figure 6: analysis of the relationship between HNRNPA2B1 expression and immune cell infiltration in tumor microenvironment. (a) The correlation between HNRNPA2B1 and infiltration level of IMMUNE cells using xCELL database; and (b) the correlation between HNRNPA2B1 and infiltration level of IMMUNE cells using CIBERSOFT database. Figure 7: correlation analysis between ESTIMATE scores and HNRNPA2B1 expression. ESTIMATEscore (a–c): (a) TARGET-ALL, TARGET-NB, TARGET-WT, TCGA-ESCA, and TCGA-GBM; (b) TCGA-LGGGBM, TCGA-KIRP, TCGA-LAML, TCGA-SARC, and TCGA-SKCM; and (c) TCGA-TGCT, TCGA-UCEC; Immunescore (d–f): (d) TARGET-ALL, TARGET-NB, TARGET-WT, TCGA-CESC, and TCGA-CHOL; (e) TCGA-ESCA, TCGA-GBM, TCGA-LGGGBM, TCGA-KIRP, and TCGA-SARC; and (f) TCGA-SKCM, TCGA-TGCT, TCGA-THCA, and TCGA-UCEC; and Stromalscore (g, h): (g) TARGET-NB, TARGET-WT, TCGA-ESCA, TCGA-GBM, and TCGA-LGGGBM, and (h) TCGA-LAML, TCGA-SARC, TCGA-SKCM, TCGA-TGCT, TCGA-THYM. Figure 8: GSEA of HNRNPA2B1 in pan-cancer. (a) ACC; (b) COAD; (c) GBM; (d) LGG; (e) OV; (f) STAD; (g) LIHC; (h) LUAD; (i) SKCM; and (j) UCS. Figure 9: the PPI network of the Top 15 hub genes was visualized by STRING database. (a) Breast cancer; (b) colorectal cancer; (c) gastric cancer; and (d) lung cancer.

Supplementary 2 Table 1: relationship between HNRNPA2B1 expression and OS of each cancer.

Supplementary 3 Table 2: relationship between HNRNPA2B1 expression and DSS of each cancer.

Supplementary 4 Table 3: relationship between HNRNPA2B1 expression and DFI of each cancer.

Supplementary 5 Table 4: relationship between HNRNPA2B1 expression and PFI of each cancer.
