# Supplementary material for: The Potential Impact of HNRNPA2B1 on Human Cancers Prognosis and Immune Microenvironment
Source: J Immunol Res. 2024 Sep 5;2024:5515307. doi: 10.1155/2024/5515307 (PMC11392580; doi:10.1155/2024/5515307)
Supplement: Supplementary 1 — Figure 1: immunohistochemical results from our hospital: (a) ESCA, up: tumor, down: normal; (b) STAD, up: tumor, down: normal; (c) COAD, up: tumor, down: normal; (d) READ, up: tumor, down: normal; (e) LIHC, up: tumor, down: normal; (f) LUSC, up: tumor, down: normal; (g) KIRC, up: tumor, down: normal; (h) BRCA, up: tumor, down: normal; (i) CESC, up: tumor, down: normal; (j) LGG, up: tumor, down: normal; (k) BLCA, up: tumor, down: normal; (l) DLBC, up: tumor, down: normal; (m) HNSC, up: tumor, down: normal; (n) GBM, up: tumor, down: normal; (o) PRAD, up: tumor, down: normal; (p) PAAD, up: tumor, down: normal; (q) endometrial cancer, up: tumor, down: normal; (r) melanoma, up: tumor, down: normal; and (s) ovarian cancer, up: tumor, down: normal. Figure 2: pan-cancer HNRNPA2B1 expression in different clinical stages. (a–h) Pan-cancer differential expression of HNRNPA2B1 in clinical stages in indicated tumor types from TCGA database. (a) ACC; (b) LUSC; (c) TGCT; (d) LIHC; (e) CESC; (f) OV; (g) KIPAN; and (h) LUAD. Figure 3: univariate Cox regression analysis of HNRNPA2B1. The forest plot shows the univariate Cox regression results of HNRNPA2B1 on pan-cancer survival (a–d). (a) OS; (b) DSS; (c) DFI; and (d) PFI. Figure 4: Kaplan–Meier survival of HNRNPA2B1 expression. OS: (a–g), (a) TARGET-LAML, (b) TCGA-LAML, (c) TCGA-LGG, (d) TCGA-PRAD, (e) TCGA-SARC, (f) TCGA-STAD, and (g) TCGA-UCEC; DSS: (h–j), (h) TCGA-LGG, (i) TCGA-SARC, and (j) TCGA-PRAD; DFI: (k–m), (k) TCGA-COAD, (l) TCGA-KIPAN, and (m) TCGA-OV; PFI: (n–q), (n) TCGA-LGG, (o) TCGA-CESC, (p) TCGA-PRAD, and (q) TCGA-UVM. Figure 5: association of HNRNPA2B1 expression with mutation landscape, different clinical stages. (a) LGG; (b) OV; (c) PRAD; (d) KIRP; (e) LAML; and (f) different clinical stages. ∗p < 0.05; ∗∗p < 0.01; ∗∗∗p < 0.001. Figure 6: analysis of the relationship between HNRNPA2B1 expression and immune cell infiltration in tumor microenvironment. (a) The correlation between HNRNPA2B1 and infiltration level o [file 5515307.f1.docx]

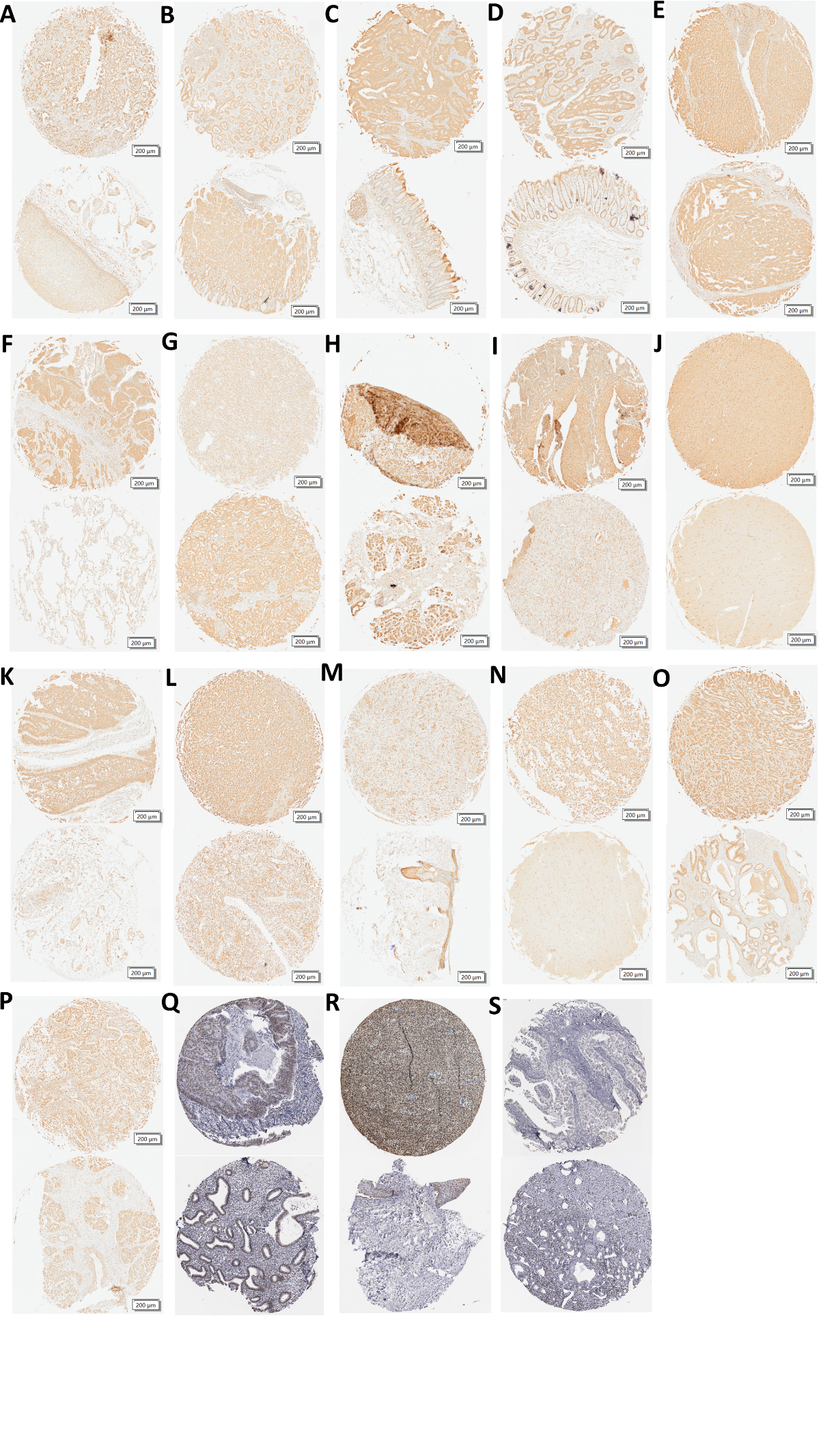


Figure S1. Immunohistochemical results from our hospital: A. ESCA, up: tumor, down: normal; B. STAD, up: tumor, down: normal; C. COAD, up: tumor, down: normal; D. READ, up: tumor, down: normal; E. LIHC, up: tumor, down: normal; F. LUSC, up: tumor, down: normal; G. KIRC, up: tumor, down: normal; H. BRCA, up: tumor, down: normal; I. CESC, up: tumor, down: normal; J. LGG, up: tumor, down: normal; K. BLCA, up: tumor, down: normal; L. DLBC, up: tumor, down: normal; M. HNSC, up: tumor, down: normal; N. GBM, up: tumor, down: normal; O. PRAD, up: tumor, down: normal; P. PAAD, up: tumor, down: normal; Q. endometrial cancer, up: tumor, down: normal; R. melanoma, up: tumor, down: normal; S. ovarian cancer, up: tumor, down: normal.


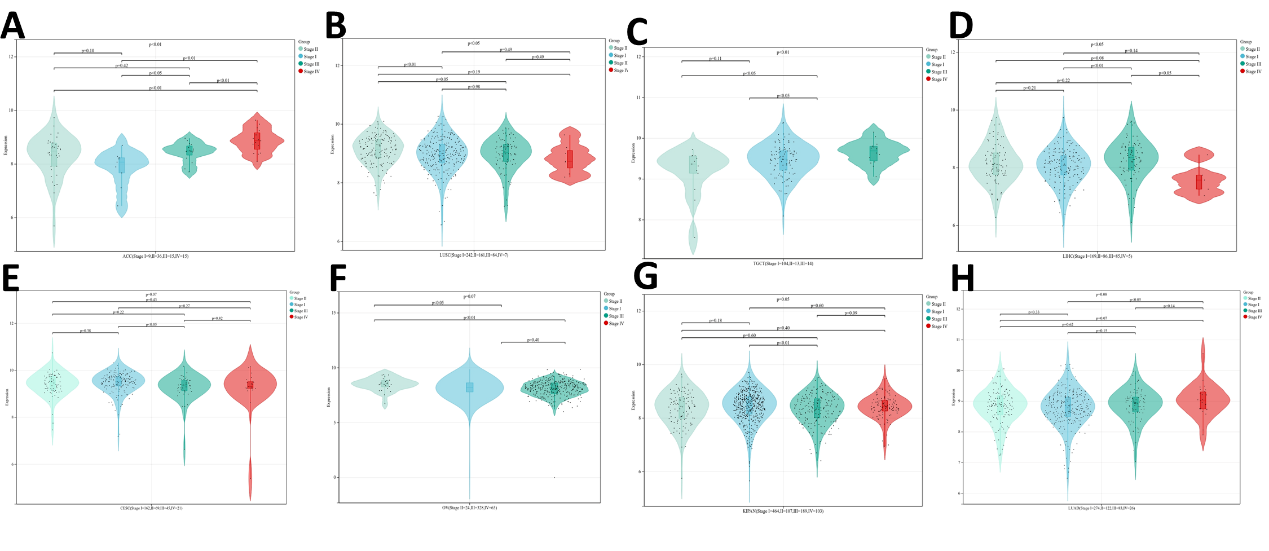


Figure S2. Pan-cancer HNRNPA2B1 expression in different Clinical stages. (A–H), Pan-cancer differential expression of HNRNPA2B1 in Clinical stages in indicated tumor types from TCGA database. A. ACC; B. LUSC; C. TGCT; D. LIHC; E. CESC; F. OV; G. KIPAN; H. LUAD.


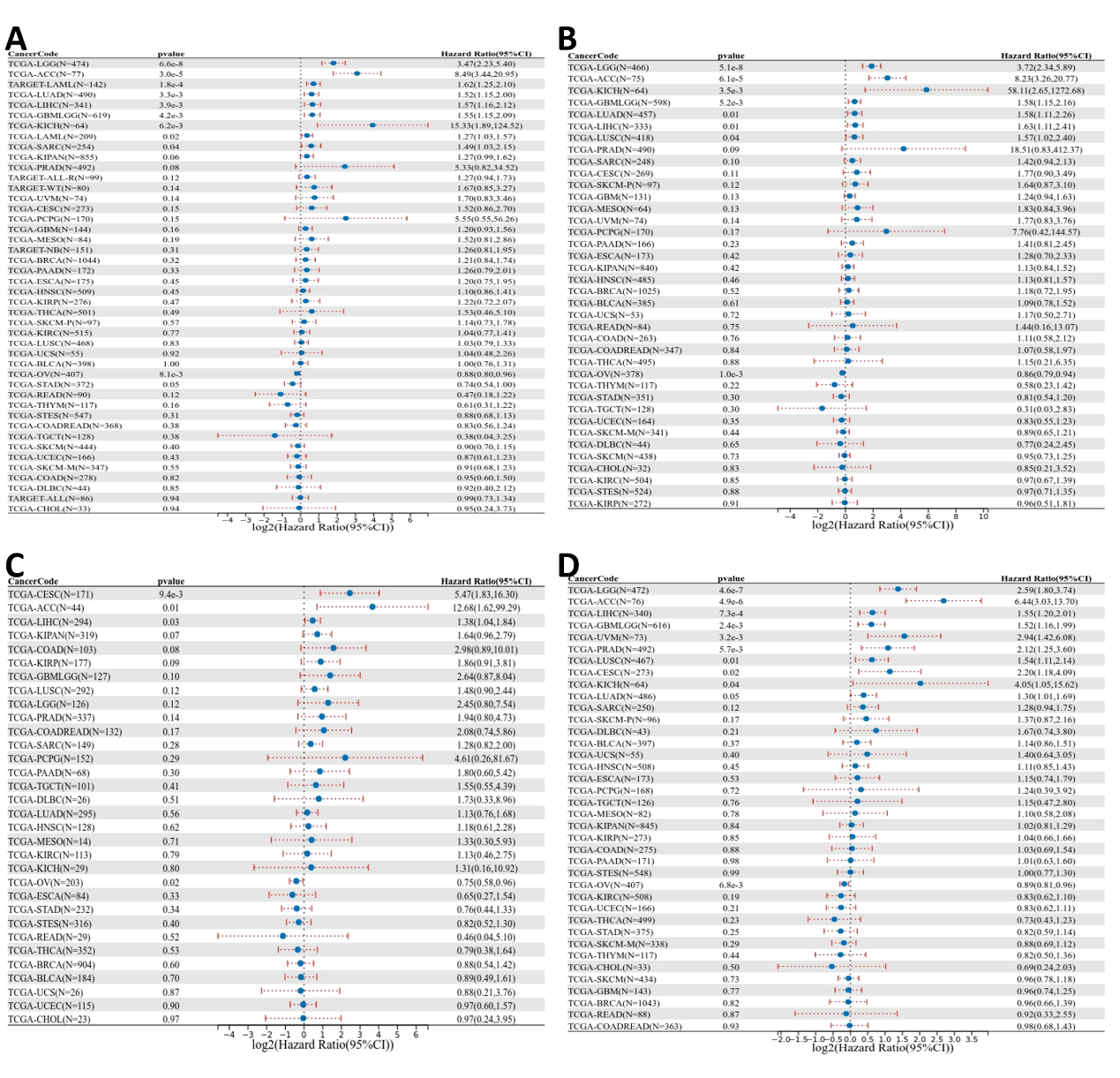


Figure S3. Univariate Cox regression analysis of HNRNPA2B1. The forest plot shows the univariate cox regression results of HNRNPA2B1 on pan-cancer survival (A-D). A. OS; B. DSS; C. DFI; D. PFI.


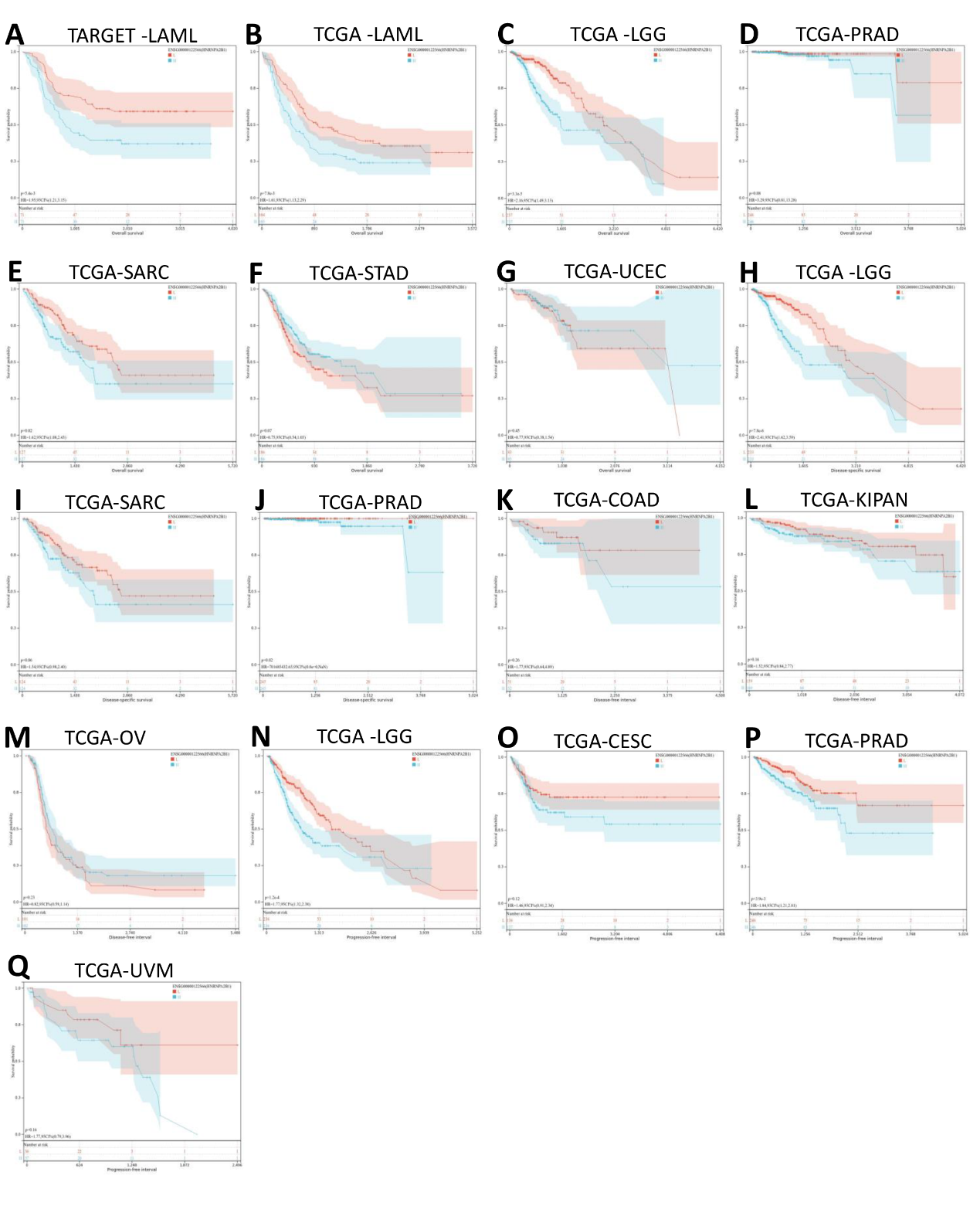
Figure S4. Kaplan-Meier survival of HNRNPA2B1 expression. OS: (A-G), A. TARGET-LAML, B. TCGA-LAML, C. TCGA-LGG, D. TCGA-PRAD, E. TCGA-SARC, F. TCGA-STAD, G. TCGA-UCEC; DSS: (H-J), H. TCGA-LGG, I. TCGA-SARC, J. TCGA-PRAD; DFI: (K-M), K. TCGA-COAD, L. TCGA-KIPAN, M. TCGA-OV; PFI: (N-Q), N. TCGA-LGG, O. TCGA-CESC, P. TCGA-PRAD, Q. TCGA-UVM.


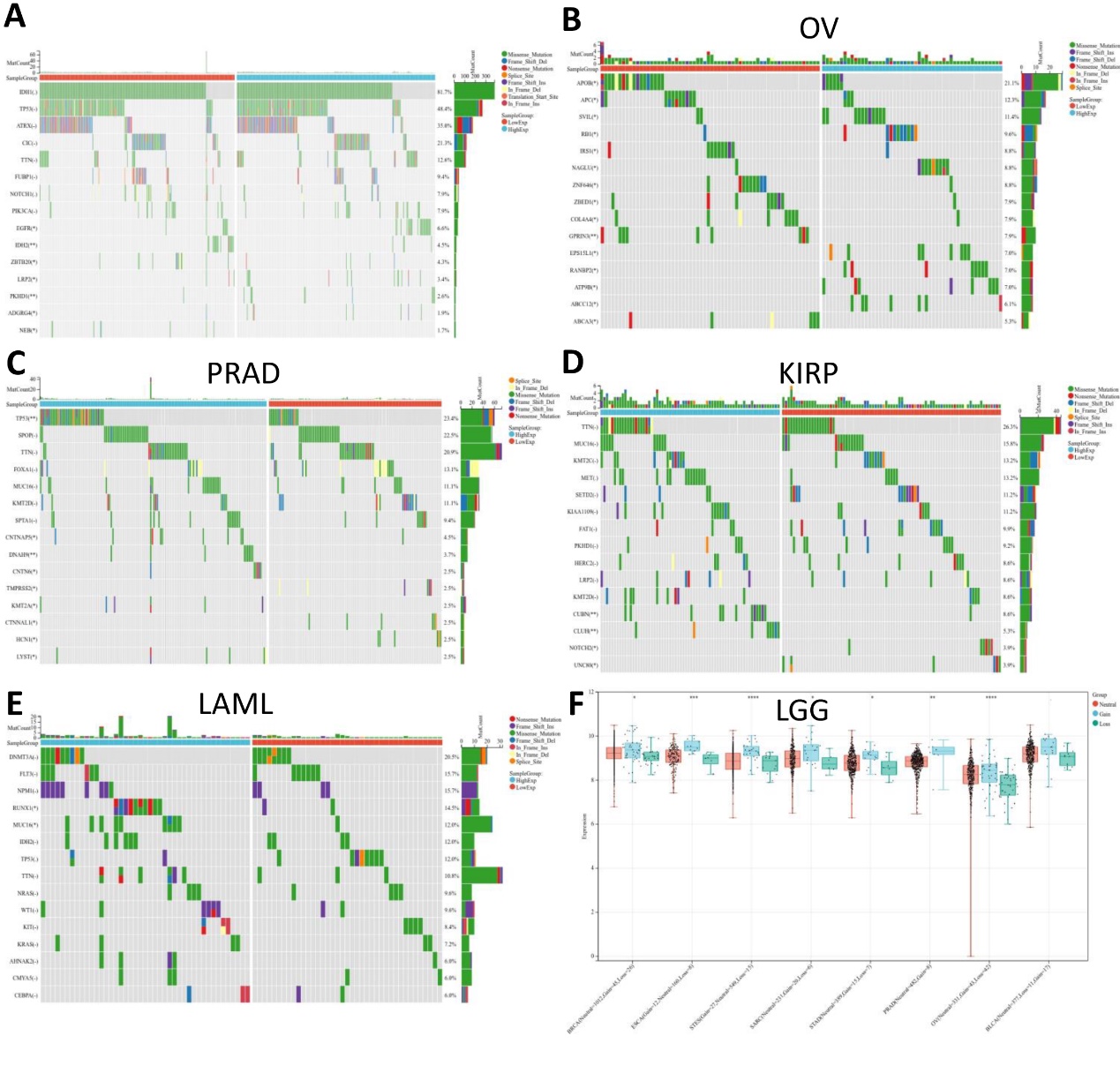


Figure S5. Association of HNRNPA2B1 expression with mutation landscape, different clinical stages. A. LGG: B. OV; C. PRAD; D. KIRP; E. LAML; F. different clinical stages. *p < 0.05; **p < 0.01; ***p < 0.001.


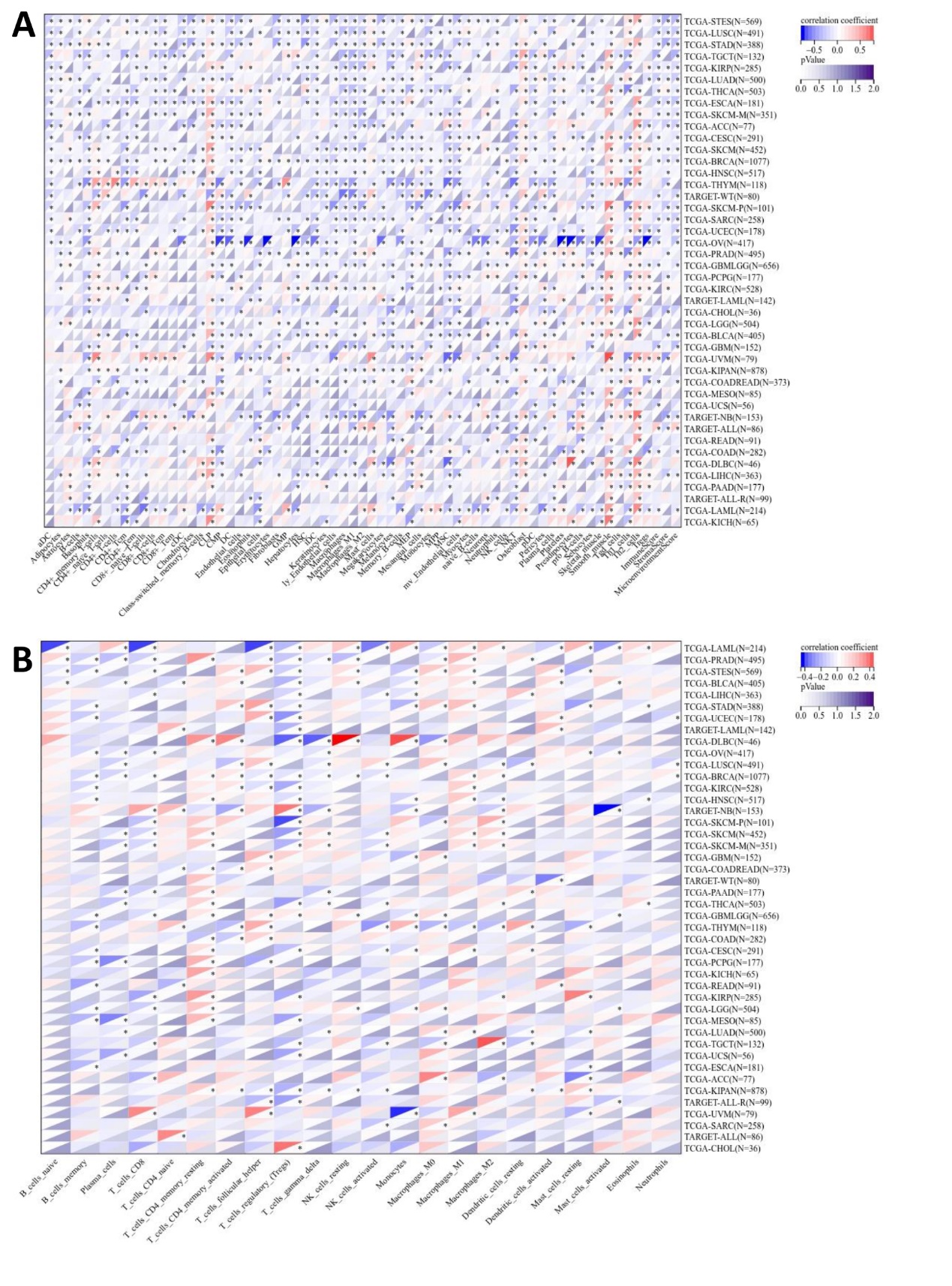


Figure S6. Analysis of the relationship between HNRNPA2B1 expression and immune cell infiltration in tumor microenviroment. A. The correlation between HNRNPA2B1 and infiltration level of IMMUNE cells using xCELL database; B. The correlation between HNRNPA2B1 and infiltration level of IMMUNE cells using CIBERSOFT database.


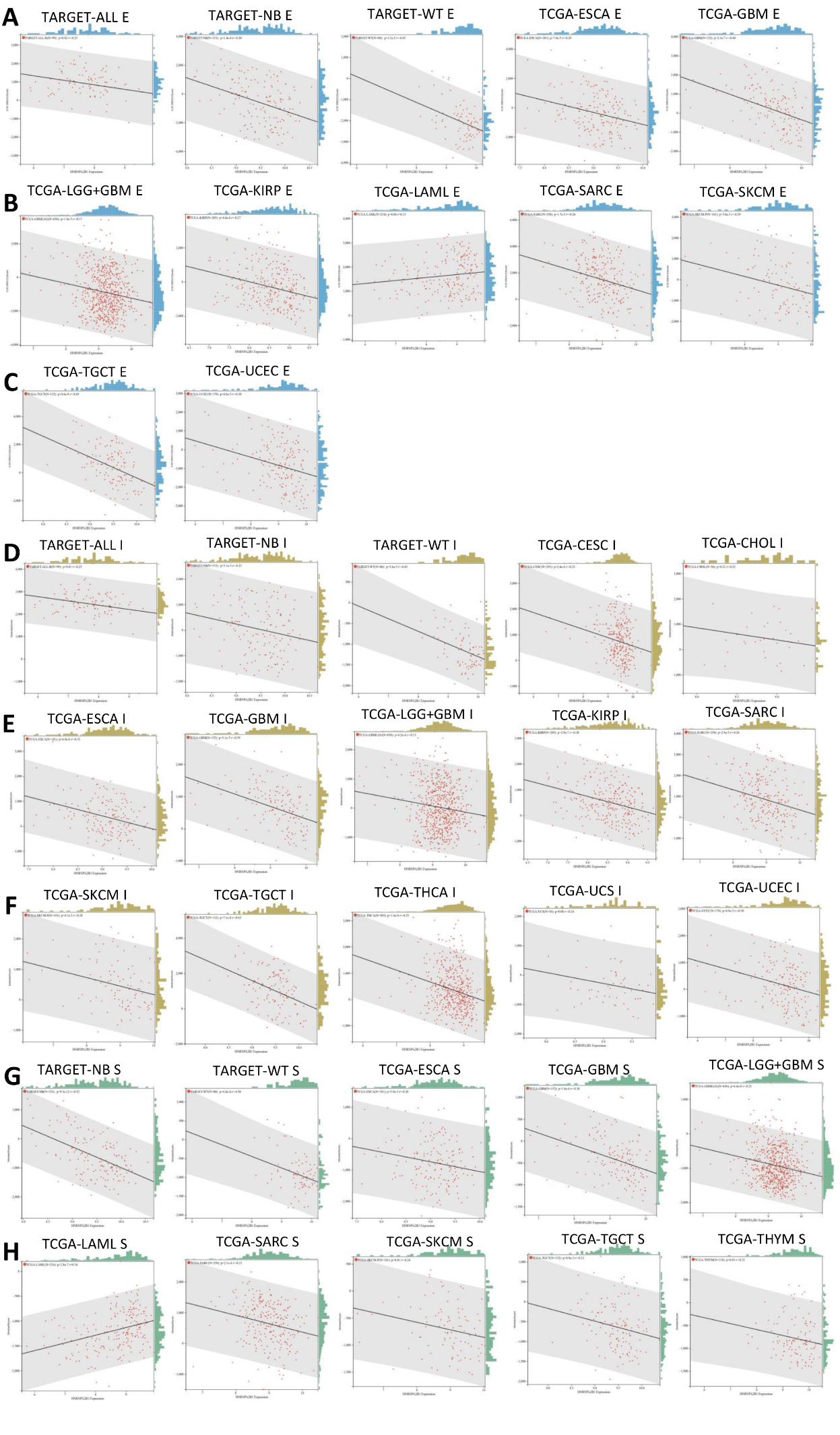
Figure S7. Correlation analysis between ESTIMATE scores and HNRNPA2B1 expression. ESTIMATEscore (A-C), A. TARGET-ALL, TARGET-NB, TARGET-WT, TCGA-ESCA, TCGA-GBM, B. TCGA-LGGGBM, TCGA-KIRP, TCGA-LAML, TCGA-SARC, TCGA-SKCM, C. TCGA-TGCT, TCGA-UCEC; Immunescore (D-F), D. TARGET-ALL, TARGET-NB, TARGET-WT, TCGA-CESC, TCGA-CHOL, E. TCGA-ESCA, TCGA-GBM, TCGA-LGGGBM, TCGA-KIRP, TCGA-SARC, F. TCGA-SKCM, TCGA-TGCT, TCGA-THCA, TCGA-UCEC; Stromalscore (G-H), G. TARGET-NB, TARGET-WT, TCGA-ESCA, TCGA-GBM, TCGA-LGGGBM, H. TCGA-LAML, TCGA-SARC, TCGA-SKCM, TCGA-TGCT, TCGA-THYM.


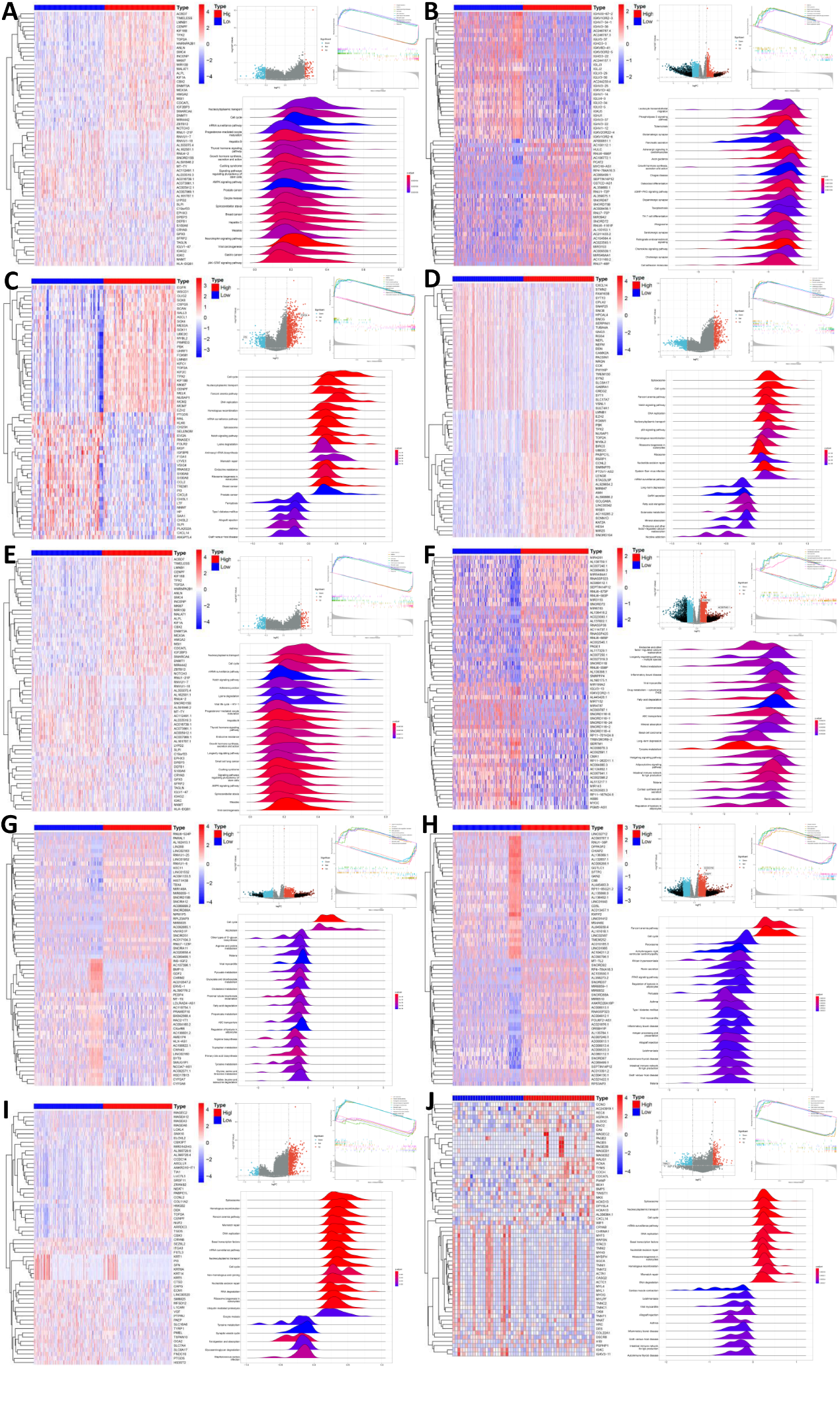


Figure S8. GSEA of HNRNPA2B1 in pan-cancer. A. ACC; B. COAD; C. GBM; D. LGG; E. OV; F. STAD; G. LIHC; H. LUAD; I. SKCM; J. UCS.


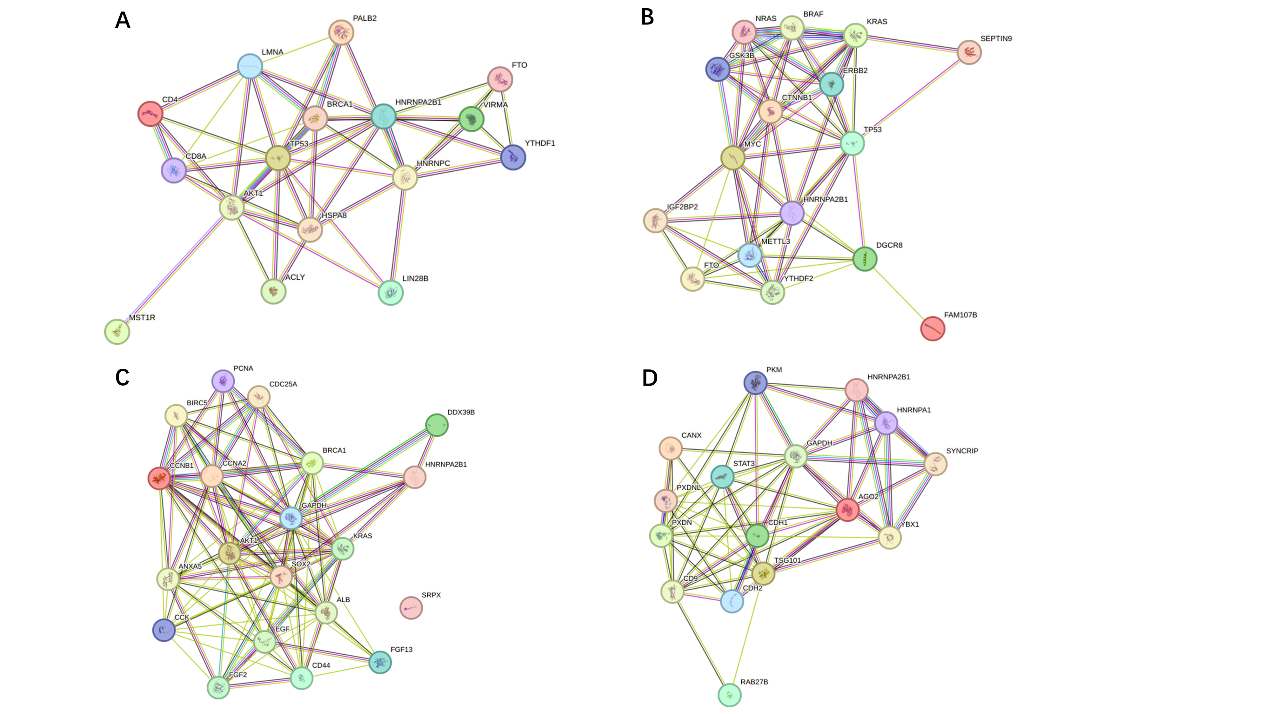


Figure S9. The PPI network of the top 15 hub genes was visualized by STRING database. A. Breast cancer; B. Colorectal cancer; C. Gastric cancer; D. Lung cancer.
