# Supplementary material for: The Potential Impact of HNRNPA2B1 on Human Cancers Prognosis and Immune Microenvironment
Source: J Immunol Res. 2024 Sep 5;2024:5515307. doi: 10.1155/2024/5515307 (PMC11392580; doi:10.1155/2024/5515307)
Supplement: Supplementary 2 — Table 1: relationship between HNRNPA2B1 expression and OS of each cancer. [file 5515307.f2.pdf]

BP

term description	observed (	backgrour	strength	FDR
Regulation of mRNA metabolic process	7	302	1.46	3.45E-05
Positive regulation of gene expression	9	1146	0.99	0.00049
Regulation of RNA stability	5	176	1.54	0.0013
Positive regulation of macromolecule metabolic process	12	3533	0.62	0.0039
Negative regulation of gene expression	7	899	0.98	0.0081
Regulation of nucleobase-containing compound metab	12	4074	0.56	0.0081
Positive regulation of cellular metabolic process	11	3114	0.64	0.0081
Regulation of DNA metabolic process	6	541	1.14	0.0081
Positive regulation of nitrogen compound metabolic pr	11	3166	0.63	0.0081
Negative regulation of organelle organization	5	351	1.24	0.0099
Nucleobase-containing compound metabolic process	10	2722	0.66	0.011
Negative regulation of macromolecule metabolic proce	10	2760	0.65	0.011
Negative regulation of mitochondrion organization	3	54	1.84	0.011
Cellular nitrogen compound metabolic process	11	3463	0.59	0.011
Regulation of mRNA stability	4	164	1.48	0.011
Cellular metabolic process	14	6568	0.42	0.011
Positive regulation of nucleobase-containing compoun	9	2056	0.73	0.011
Negative regulation of cellular component organization	6	691	1.03	0.011
Regulation of release of cytochrome c from mitochondr	3	47	1.9	0.011
Nucleic acid metabolic process	9	2203	0.7	0.011
Negative regulation of cellular metabolic process	9	2265	0.69	0.012
Regulation of cellular metabolic process	13	5681	0.45	0.0125
Regulation of RNA metabolic process	11	3759	0.56	0.0125
Regulation of cellular catabolic process	6	789	0.97	0.0128
Regulation of nitrogen compound metabolic process	13	5734	0.45	0.0128
Negative regulation of nitrogen compound metabolic p	9	2403	0.66	0.0156
Regulation of primary metabolic process	13	5899	0.43	0.0163
Nucleocytoplasmic transport	4	248	1.3	0.0177
RNA destabilization	3	79	1.67	0.0177
Positive regulation of RNA metabolic process	8	1845	0.73	0.0177
Cellular response to granulocyte macrophage colony-st	2	10	2.39	0.0177
Positive regulation of histone H3-K9 methylation	2	11	2.35	0.0188
Positive regulation of biological process	13	6207	0.41	0.0226
Regulation of macromolecule metabolic process	13	6249	0.41	0.0239
Negative regulation of extrinsic apoptotic signaling path	3	97	1.58	0.0241
Regulation of telomere maintenance	3	103	1.55	0.028
Negative regulation of small molecule metabolic proces	3	104	1.55	0.0282
Negative regulation of miRNA maturation	2	16	2.19	0.0306
mRNA metabolic process	5	611	1	0.0308
Positive regulation of histone modification	3	111	1.52	0.0308
Positive regulation of protein modification process	6	1018	0.86	0.0308
Positive regulation of protein metabolic process	7	1512	0.76	0.0308
Negative regulation of macromolecule biosynthetic pro	7	1532	0.75	0.0314
Protein import into nucleus	3	115	1.51	0.0321
RNA metabolic process	7	1550	0.75	0.0326
Enzyme-linked receptor protein signaling pathway	5	641	0.98	0.0337
Cellular response to stress	7	1572	0.74	0.0338
Nitrogen compound metabolic process	13	6643	0.38	0.0353
Negative regulation of cellular biosynthetic process	7	1592	0.73	0.0353
Establishment of protein localization to organelle	4	335	1.17	0.0353
Positive regulation of mRNA metabolic process	3	123	1.48	0.0353
Regulation of DNA biosynthetic process	3	124	1.47	0.0353
Negative regulation of release of cytochrome c from mi	2	21	2.07	0.0366
Cellular response to decreased oxygen levels	3	136	1.43	0.0391
Regulation of apoptotic signaling pathway	4	365	1.13	0.0395
RNA splicing	4	370	1.12	0.0406

Cytokine-mediated signaling pathway	4	369	1.13	0.0406
Negative regulation of cellular process	11	4736	0.46	0.0406
Regulation of mRNA processing	3	140	1.42	0.0406
Cellular response to cytokine stimulus	5	711	0.94	0.0406
Macromolecule metabolic process	12	5781	0.41	0.0422
Regulation of organelle organization	6	1190	0.79	0.0448
Regulation of cellular component organization	8	2365	0.62	0.0448
Regulation of gene expression	11	4899	0.44	0.0487
T cell apoptotic process	2	29	1.93	0.0499

FDR: false discovery rate

MF

term description	observed	backgrour	strength	FDR
N6-methyladenosine-containing RNA binding	3	10	2.57	0.00062
mRNA binding	5	326	1.28	0.009
MHC protein complex binding	3	35	2.02	0.009
RNA binding	8	1672	0.77	0.0233

CC

term description	observed	backgrour	strength	FDR
Nucleoplasm	12	4169	0.55	0.0128
Nuclear body	6	833	0.95	0.0157
Intracellular organelle lumen	13	5660	0.45	0.0157
T cell receptor complex	2	14	2.25	0.0215
Nuclear matrix	3	128	1.46	0.0386

KEGG

term description	observed	backgrour	strength	FDR
Platinum drug resistance	3	70	1.72	0.0068
Antigen processing and presentation	3	64	1.76	0.0068
T cell receptor signaling pathway	3	100	1.57	0.0083
Apoptosis	3	131	1.45	0.0115
Yersinia infection	3	124	1.47	0.0115
Measles	3	137	1.43	0.0115
Breast cancer	3	146	1.4	0.0115
Homologous recombination	2	38	1.81	0.0189
Primary immunodeficiency	2	37	1.82	0.0189
Human T-cell leukemia virus 1 infection	3	210	1.25	0.0211
Fanconi anemia pathway	2	51	1.68	0.0254
Endometrial cancer	2	58	1.63	0.0298
Longevity regulating pathway - multiple species	2	61	1.61	0.0303
MAPK signaling pathway	3	286	1.11	0.0347
Colorectal cancer	2	82	1.48	0.0347
Pancreatic cancer	2	71	1.54	0.0347
Glioma	2	71	1.54	0.0347
Melanoma	2	72	1.53	0.0347
Chronic myeloid leukemia	2	75	1.52	0.0347
Non-small cell lung cancer	2	68	1.56	0.0347
Central carbon metabolism in cancer	2	68	1.56	0.0347
Endocrine resistance	2	94	1.42	0.0354
PI3K-Akt signaling pathway	3	349	1.02	0.0354
Longevity regulating pathway	2	87	1.45	0.0354
Hematopoietic cell lineage	2	90	1.44	0.0354
Prostate cancer	2	97	1.4	0.0354
Small cell lung cancer	2	92	1.43	0.0354
PD-L1 expression and PD-1 checkpoint pathway in	2	87	1.45	0.0354
Toxoplasmosis	2	103	1.38	0.0372

Neurotrophin signaling pathway	2	112	1.34	0.0422
Sphingolipid signaling pathway	2	116	1.33	0.0437
Thyroid hormone signaling pathway	2	120	1.31	0.0452
