# Supplementary material for: The Potential Impact of HNRNPA2B1 on Human Cancers Prognosis and Immune Microenvironment
Source: J Immunol Res. 2024 Sep 5;2024:5515307. doi: 10.1155/2024/5515307 (PMC11392580; doi:10.1155/2024/5515307)
Supplement: Supplementary 3 — Table 2: relationship between HNRNPA2B1 expression and DSS of each cancer. [file 5515307.f3.pdf]

BP

term description	observed	backgrour	strength	FDR
Positive regulation of gene expression	10	1146	1.03	4.20E-05
Negative regulation of gene expression	9	899	1.09	4.20E-05
Regulation of cellular macromolecule bios	8	539	1.26	4.20E-05
Regulation of cell differentiation	10	1582	0.89	0.00023
Regulation of cellular amide metabolic pr	7	516	1.22	0.00026
Regulation of telomerase activity	4	51	1.98	0.00026
Primary miRNA processing	3	13	2.45	0.00048
T cell differentiation in thymus	4	63	1.89	0.00048
Negative regulation of cellular macromole	5	189	1.51	0.00061
Negative regulation of cell differentiati	7	685	1.1	0.00085
Regulation of protein metabolic process	11	2622	0.71	0.00085
MAPK cascade	5	219	1.45	0.00086
Negative regulation of macromolecule bios	9	1532	0.86	0.00086
Positive regulation of protein metabolic	9	1512	0.87	0.00086
Regulation of multicellular organismal pr	11	2749	0.69	0.00092
Regulation of translation	6	456	1.21	0.00097
Positive regulation of macromolecule meta	12	3533	0.62	0.00097
Negative regulation of cellular biosynthe	9	1592	0.84	0.00097
Gliogenesis	5	248	1.39	0.001
Regulation of cell population proliferati	9	1669	0.82	0.0011
Positive regulation of organelle organiza	6	508	1.16	0.0014
Regulation of cell development	6	506	1.16	0.0014
Positive regulation of cellular metabolic	11	3114	0.64	0.0018
Regulation of gene expression	13	4899	0.51	0.0019
Positive regulation of nitrogen compound	11	3166	0.63	0.0019
Regulation of mRNA metabolic process	5	302	1.31	0.0019
Response to light stimulus	5	314	1.29	0.002
Regulation of neuron death	5	324	1.28	0.002
Regulation of leukocyte differentiation	5	315	1.29	0.002
Regulation of multicellular organismal de	8	1389	0.85	0.002
Positive regulation of translation	4	144	1.53	0.0021
Positive regulation of biological process	14	6207	0.44	0.0023
Endothelial to hematopoietic transition	2	3	2.91	0.0024
Positive regulation of cap-independent tr	2	3	2.91	0.0024
Regulation of mRNA stability	4	164	1.48	0.003
Regulation of neurogenesis	5	368	1.22	0.003
Negative regulation of protein metabolic	7	1038	0.92	0.003
Negative regulation of macromolecule meta	10	2760	0.65	0.0031
Negative regulation of translation	4	169	1.46	0.0031
Intracellular signal transduction	8	1518	0.81	0.0031
Negative regulation of biological process	13	5313	0.48	0.0031
Glial cell differentiation	4	188	1.42	0.004
Anatomical structure morphogenesis	9	2229	0.7	0.0044
Positive regulation of cellular process	13	5584	0.46	0.0044
Negative regulation of immune system proc	5	433	1.15	0.0048
Regulation of organelle organization	7	1190	0.86	0.0051
Positive regulation of epithelial cell pr	4	207	1.38	0.0051
Regulation of cellular catabolic process	6	789	0.97	0.0053
Regulation of neuron apoptotic process	4	216	1.36	0.0055
Negative regulation of cellular process	12	4736	0.49	0.0055
Negative regulation of neuron death	4	219	1.35	0.0056
Negative regulation of nitrogen compound	9	2403	0.66	0.0064
Head development	6	823	0.95	0.0064
mRNA destabilization	3	75	1.69	0.0066
Stem cell proliferation	3	78	1.68	0.0073
Regulation of stem cell proliferation	3	81	1.66	0.008

Regulation of stem cell differentiation	3	82	1.65	0.0082
Animal organ development	10	3246	0.58	0.0088
Negative regulation of cell communication	7	1348	0.81	0.009
Negative regulation of signaling	7	1354	0.8	0.009
Negative regulation of apoptotic process	6	891	0.92	0.009
ER overload response	2	12	2.31	0.0097
Cellular response to UV	3	90	1.61	0.0097
Regulation of fibroblast proliferation	3	89	1.62	0.0097
Regulation of transferase activity	6	912	0.91	0.0097
Regulation of response to stress	7	1373	0.8	0.0097
Positive regulation of neuron death	3	92	1.6	0.0099
Cell morphogenesis involved in differenti	5	554	1.05	0.0102
Oogenesis	3	94	1.59	0.0103
Regulation of gliogenesis	3	101	1.56	0.0122
Regulation of telomere maintenance	3	103	1.55	0.0127
Positive regulation of nucleobase-contain	8	2056	0.68	0.0131
Positive regulation of intracellular sign	6	997	0.87	0.0134
Negative regulation of leukocyte differen	3	107	1.54	0.0135
Central nervous system development	6	1035	0.85	0.0158
Stem cell population maintenance	3	114	1.51	0.0158
Apoptotic process	6	1041	0.85	0.0161
RNA metabolic process	7	1550	0.75	0.0162
Enzyme-linked receptor protein signaling	5	641	0.98	0.0163
Regulation of hematopoietic stem cell dif	2	18	2.14	0.0163
Negative regulation of type I interferon-	2	19	2.11	0.0172
Regulation of biological quality	10	3654	0.53	0.0172
Hemopoiesis	5	655	0.97	0.0175
Response to abiotic stimulus	6	1107	0.82	0.0201
Regulation of signal transduction	9	2978	0.57	0.0216
Branching morphogenesis of an epithelial	3	134	1.44	0.0216
Regulation of cellular metabolic process	12	5681	0.42	0.0218
Positive regulation of mesenchymal cell p	2	23	2.03	0.0225
Regulation of apoptotic signaling pathway	4	365	1.13	0.0225
Circadian rhythm	3	139	1.42	0.0227
T cell lineage commitment	2	24	2.01	0.0228
Cell population proliferation	5	712	0.94	0.0228
Regulation of nitrogen compound metabolic	12	5734	0.41	0.0228
Regulation of cellular response to stress	5	712	0.94	0.0228
Regulation of T cell activation	4	376	1.12	0.0234
System development	10	3867	0.5	0.024
Lung epithelial cell differentiation	2	25	1.99	0.024
Cell development	7	1719	0.7	0.0242
Regulation of intracellular signal transd	7	1726	0.7	0.0246
Regulation of cellular component organiza	8	2365	0.62	0.0249
Epithelial tube branching involved in lun	2	26	1.98	0.0249
Forebrain development	4	391	1.1	0.0256
Glial cell proliferation	2	27	1.96	0.0262
Regulation of primary metabolic process	12	5899	0.4	0.0271
Positive regulation of cilium assembly	2	28	1.94	0.0272
Negative regulation of stem cell differen	2	28	1.94	0.0272
Regulation of macromolecule biosynthetic	10	3980	0.49	0.0274
Brain development	5	775	0.9	0.029
Positive regulation of cellular catabolic	4	413	1.08	0.0291
Negative regulation of signal transductio	6	1252	0.77	0.0297
Regulation of cell adhesion	5	784	0.89	0.0297
Response to xenobiotic stimulus	4	422	1.07	0.0304
Transmembrane receptor protein tyrosine k	4	425	1.06	0.0311
Positive regulation of mitochondrial memb	2	32	1.89	0.0321

Positive regulation of RNA metabolic proc	7	1845	0.67	0.0321
Regulation of intrinsic apoptotic signali	2	32	1.89	0.0321
Neurogenesis	6	1290	0.76	0.0332
Regulation of cellular biosynthetic proce	10	4143	0.47	0.0349
Negative regulation of fibroblast prolife	2	34	1.86	0.0349
Positive regulation of telomerase activit	2	34	1.86	0.0349
Regulation of T cell differentiation	3	178	1.32	0.035
Regulation of glial cell proliferation	2	35	1.85	0.036
Negative regulation of cell development	3	181	1.31	0.0363
Anatomical structure development	11	5117	0.42	0.0363
Cellular response to xenobiotic stimulus	3	181	1.31	0.0363
Positive regulation of telomere maintenan	2	37	1.82	0.0379
Negative regulation of Notch signaling pa	2	38	1.81	0.0393
Regulation of synaptic plasticity	3	192	1.28	0.0401
Ras protein signal transduction	3	194	1.28	0.0408
Negative regulation of cell-cell adhesion	3	194	1.28	0.0408
Regulation of neuroblast proliferation	2	39	1.8	0.0408
Negative regulation of gliogenesis	2	41	1.78	0.044
Organelle organization	9	3470	0.5	0.0451
Positive regulation of cellular component	4	500	0.99	0.0476
Response to growth factor	4	503	0.99	0.0481
Regulation of reproductive process	3	208	1.25	0.0481
Regulation of immune system process	6	1438	0.71	0.0498

FDR: false discovery rate

MF

term description	observed	backgrour	strength	FDR
mRNA binding	6	326	1.36	0.00062
N6-methyladenosine-containing RNA binding	3	10	2.57	0.00062

KEGG

term description	observed	backgrour	strength	FDR
Endometrial cancer	8	58	2.23	4.30E-14
Breast cancer	8	146	1.83	2.37E-11
Gastric cancer	8	146	1.83	2.37E-11
Colorectal cancer	7	82	2.02	2.79E-11
Thyroid cancer	6	37	2.3	3.96E-11
Prostate cancer	7	97	1.95	5.07E-11
Bladder cancer	6	40	2.27	5.07E-11
Hepatitis C	7	157	1.74	1.09E-09
Hepatocellular carcinoma	7	161	1.73	1.15E-09
ErbB signaling pathway	6	81	1.96	1.61E-09
Proteoglycans in cancer	7	194	1.65	3.33E-09
Thyroid hormone signaling pathway	6	120	1.79	1.29E-08
Pathways in cancer	8	515	1.28	6.42E-08
Non-small cell lung cancer	5	68	1.96	6.42E-08
Central carbon metabolism in cancer	5	68	1.96	6.42E-08
Chronic myeloid leukemia	5	75	1.91	8.58E-08
EGFR tyrosine kinase inhibitor resistance	5	77	1.9	9.16E-08
Kaposi sarcoma-associated herpesvirus inf	6	187	1.6	1.12E-07
Endocrine resistance	5	94	1.82	2.13E-07
Human cytomegalovirus infection	6	217	1.53	2.40E-07
Neurotrophin signaling pathway	5	112	1.74	4.48E-07
MAPK signaling pathway	6	286	1.41	1.08E-06
Signaling pathways regulating pluripotenc	5	141	1.64	1.24E-06
Hepatitis B	5	158	1.59	2.06E-06

MicroRNAs in cancer	5	159	1.59	2.06E-06
PI3K-Akt signaling pathway	6	349	1.33	2.90E-06
Acute myeloid leukemia	4	67	1.87	3.38E-06
Pancreatic cancer	4	71	1.84	4.07E-06
Glioma	4	71	1.84	4.07E-06
Melanoma	4	72	1.84	4.07E-06
Melanogenesis	4	95	1.71	1.13E-05
Insulin signaling pathway	4	132	1.57	3.88E-05
Human papillomavirus infection	5	324	1.28	4.80E-05
mTOR signaling pathway	4	150	1.52	5.99E-05
Cellular senescence	4	150	1.52	5.99E-05
Wnt signaling pathway	4	154	1.5	6.26E-05
Alzheimer disease	5	354	1.24	6.55E-05
Chemokine signaling pathway	4	186	1.42	0.00012
Focal adhesion	4	195	1.4	0.00014
Long-term depression	3	59	1.8	0.00014
Rap1 signaling pathway	4	201	1.39	0.00015
Mitophagy - animal	3	64	1.76	0.00016
Long-term potentiation	3	63	1.77	0.00016
Human T-cell leukemia virus 1 infection	4	210	1.37	0.00016
Renal cell carcinoma	3	65	1.75	0.00016
Basal cell carcinoma	3	63	1.77	0.00016
Prolactin signaling pathway	3	68	1.73	0.00017
B cell receptor signaling pathway	3	78	1.68	0.00025
Longevity regulating pathway	3	87	1.63	0.00034
T cell receptor signaling pathway	3	100	1.57	0.0005
Serotonergic synapse	3	108	1.53	0.00061
Sphingolipid signaling pathway	3	116	1.5	0.00073
Growth hormone synthesis, secretion and a	3	117	1.5	0.00074
Cell cycle	3	120	1.49	0.00078
Natural killer cell mediated cytotoxicity	3	120	1.49	0.00078
FoxO signaling pathway	3	126	1.47	0.00086
Apoptosis	3	131	1.45	0.00095
Alcoholism	3	146	1.4	0.0013
Hippo signaling pathway	3	154	1.38	0.0014
Cushing syndrome	3	153	1.38	0.0014
Axon guidance	3	176	1.32	0.0021
Viral carcinogenesis	3	183	1.3	0.0023
Regulation of actin cytoskeleton	3	209	1.25	0.0033
Shigellosis	3	218	1.23	0.0037
VEGF signaling pathway	2	56	1.64	0.0051
Longevity regulating pathway - multiple s	2	61	1.61	0.006
GnRH secretion	2	63	1.59	0.0063
Fc epsilon RI signaling pathway	2	65	1.58	0.0065
Adherens junction	2	69	1.55	0.0072
Platinum drug resistance	2	70	1.55	0.0073
Bacterial invasion of epithelial cells	2	70	1.55	0.0073
Gap junction	2	87	1.45	0.0108
GnRH signaling pathway	2	87	1.45	0.0108
PD-L1 expression and PD-1 checkpoint path	2	87	1.45	0.0108
Small cell lung cancer	2	92	1.43	0.0116
Progesterone-mediated oocyte maturation	2	95	1.41	0.0122
AGE-RAGE signaling pathway in diabetic co	2	96	1.41	0.0122
Choline metabolism in cancer	2	95	1.41	0.0122
C-type lectin receptor signaling pathway	2	101	1.39	0.0132
Cholinergic synapse	2	109	1.35	0.015
Relaxin signaling pathway	2	126	1.29	0.0196
Fluid shear stress and atherosclerosis	2	129	1.28	0.0203

Autophagy - animal	2	131	1.27	0.0206
Apelin signaling pathway	2	133	1.27	0.021
Estrogen signaling pathway	2	133	1.27	0.021
Measles	2	137	1.25	0.0217
Phospholipase D signaling pathway	2	147	1.22	0.0245
Oxytocin signaling pathway	2	147	1.22	0.0245
Transcriptional misregulation in cancer	2	171	1.16	0.032
Epstein-Barr virus infection	2	192	1.11	0.0394
Human immunodeficiency virus 1 infection	2	203	1.08	0.0433
Salmonella infection	2	209	1.07	0.0453
