# Supplementary material for: The Potential Impact of HNRNPA2B1 on Human Cancers Prognosis and Immune Microenvironment
Source: J Immunol Res. 2024 Sep 5;2024:5515307. doi: 10.1155/2024/5515307 (PMC11392580; doi:10.1155/2024/5515307)
Supplement: Supplementary 4 — Table 3: relationship between HNRNPA2B1 expression and DFI of each cancer. [file 5515307.f4.pdf]

BP

term description	observed (	backgrour	strength	FDR
Regulation of catalytic activity	14	2370	0.76	4.10E-05
Regulation of transferase activity	10	912	1.03	4.50E-05
Regulation of protein phosphorylation	10	1108	0.95	0.00019
Positive regulation of macromolecule metabolic process	15	3533	0.62	0.00019
Positive regulation of cellular metabolic process	14	3114	0.65	0.00019
Regulation of protein modification process	11	1560	0.84	0.00019
Positive regulation of cellular process	17	5584	0.48	0.00019
Positive regulation of nitrogen compound metabolic proc	14	3166	0.64	0.00019
Regulation of protein metabolic process	13	2622	0.69	0.00019
Positive regulation of transferase activity	8	586	1.13	0.00019
Regulation of cell cycle	10	1108	0.95	0.00019
Positive regulation of cell cycle process	6	251	1.37	0.00019
Regulation of cell cycle checkpoint	4	49	1.91	0.00019
Regulation of cell cycle phase transition	7	431	1.2	0.00019
Positive regulation of cell cycle phase transition	5	115	1.63	0.00019
Regulation of intracellular signal transduction	11	1726	0.8	0.00019
Regulation of cell cycle process	8	716	1.04	0.00021
Positive regulation of protein modification process	9	1018	0.94	0.00021
Positive regulation of protein phosphorylation	8	747	1.02	0.00024
G2/M transition of mitotic cell cycle	4	58	1.83	0.00026
Positive regulation of DNA metabolic process	6	304	1.29	0.00028
Regulation of kinase activity	8	778	1.01	0.00029
Regulation of DNA metabolic process	7	541	1.11	0.00038
Negative regulation of biological process	16	5313	0.47	0.00059
Regulation of protein serine/threonine kinase activity	6	386	1.18	0.00079
Negative regulation of cellular process	15	4736	0.49	0.00097
Positive regulation of mitotic cell cycle phase transition	4	92	1.63	0.00099
Regulation of protein kinase activity	7	663	1.02	0.0011
Regulation of cellular metabolic process	16	5681	0.44	0.0012
Regulation of nitrogen compound metabolic process	16	5734	0.44	0.0014
Negative regulation of cell death	8	1016	0.89	0.0014
Regulation of cyclin-dependent protein serine/threonine	4	111	1.55	0.0018
Regulation of signal transduction	12	2978	0.6	0.002
Regulation of primary metabolic process	16	5899	0.43	0.002
Regulation of programmed cell death	9	1492	0.77	0.0022
Regulation of mitotic cell cycle	6	493	1.08	0.0023
Positive regulation of kinase activity	6	494	1.08	0.0023
Regulation of cellular process	20	11025	0.25	0.0028
Mitotic cell cycle process	6	537	1.04	0.0035
Regulation of macromolecule metabolic process	16	6249	0.4	0.0036
Regulation of response to stimulus	13	3931	0.51	0.0041
Positive regulation of response to stimulus	10	2131	0.66	0.0041
Regulation of cell population proliferation	9	1669	0.73	0.0043
Cellular response to stimulus	16	6357	0.39	0.0043
Negative regulation of apoptotic process	7	891	0.89	0.0046
Regulation of mitotic cell cycle phase transition	5	332	1.17	0.0046
Positive regulation of deoxyribonuclease activity	2	5	2.6	0.005
Positive regulation of cell population proliferation	7	945	0.86	0.0062
Regulation of epithelial cell proliferation	5	366	1.13	0.0063
Glial cell differentiation	4	188	1.32	0.008
Positive regulation of intracellular signal transduction	7	997	0.84	0.008
Regulation of hydrolase activity	7	1011	0.83	0.0085
Cerebellar granule cell precursor proliferation	2	8	2.39	0.0089
Positive regulation of cerebellar granule cell precursor pr	2	8	2.39	0.0089
Response to stimulus	17	7835	0.33	0.0089
Positive regulation of epithelial tube formation	2	8	2.39	0.0089

Regulation of endopeptidase activity	5	414	1.08	0.0093
Regulation of apoptotic process	8	1462	0.73	0.0094
Regulation of cysteine-type endopeptidase activity involv	4	205	1.28	0.0094
Positive regulation of protein kinase activity	5	416	1.07	0.0094
Gland development	5	419	1.07	0.0094
Cell population proliferation	6	712	0.92	0.01
MAPK cascade	4	219	1.26	0.011
Hyaluronan catabolic process	2	10	2.29	0.011
Negative regulation of cysteine-type endopeptidase activ	3	75	1.6	0.011
Intracellular signal transduction	8	1518	0.72	0.0112
Response to radiation	5	444	1.05	0.0113
Response to abiotic stimulus	7	1107	0.79	0.0113
Positive regulation of signal transduction	8	1525	0.71	0.0113
Regulation of proteolysis	6	739	0.9	0.0113
Positive regulation of attachment of spindle microtubules	2	11	2.25	0.0116
Positive regulation of protein serine/threonine kinase acti	4	226	1.24	0.0116
Positive regulation of nucleobase-containing compound	9	2056	0.63	0.0128
Negative regulation of endopeptidase activity	4	240	1.22	0.0139
Cellular component organization	14	5436	0.4	0.0141
Regulation of organelle organization	7	1190	0.76	0.0159
Response to growth factor	5	503	0.99	0.0172
Positive regulation of endothelial cell proliferation	3	96	1.49	0.0179
Response to stress	11	3358	0.51	0.0179
Neural precursor cell proliferation	3	98	1.48	0.0185
Regulation of G2/M transition of mitotic cell cycle	3	99	1.47	0.0189
Cell division	5	527	0.97	0.0198
Adenohypophysis development	2	17	2.06	0.0209
Microtubule cytoskeleton organization	5	542	0.96	0.0222
Organelle organization	11	3470	0.49	0.0222
Positive regulation of endothelial cell migration	3	107	1.44	0.0222
Mammary gland epithelial cell differentiation	2	18	2.04	0.0225
Cell migration involved in sprouting angiogenesis	2	20	1.99	0.0267
Regulation of mitotic nuclear division	3	118	1.4	0.0283
Regulation of mitotic cell cycle spindle assembly checkpo	2	21	1.97	0.0288
Response to light stimulus	4	314	1.1	0.0302
Regulation of response to DNA damage stimulus	4	316	1.1	0.0307
Regulation of DNA biosynthetic process	3	124	1.38	0.0312
Phosphate-containing compound metabolic process	8	1877	0.62	0.0332
Phosphorylation	6	966	0.79	0.0332
Mammary gland development	3	128	1.36	0.0332
Chromosome organization	6	968	0.79	0.0332
Cellular response to abiotic stimulus	4	325	1.08	0.0332
Branching morphogenesis of an epithelial tube	3	134	1.34	0.0363
Regulation of DNA replication	3	136	1.34	0.0374
Positive regulation of macromolecule biosynthetic proces	8	1935	0.61	0.0382
Regulation of DNA damage checkpoint	2	27	1.86	0.0395
Osteoblast differentiation	3	141	1.32	0.0399
Negative regulation of organelle organization	4	351	1.05	0.0399
Positive regulation of G2/M transition of mitotic cell cycle	2	28	1.85	0.0412
Regulation of apoptotic signaling pathway	4	365	1.03	0.0449
Response to UV	3	150	1.29	0.0459
Negative regulation of lipid transport	2	30	1.82	0.0459
Regulation of extrinsic apoptotic signaling pathway	3	153	1.29	0.0477
Regeneration	3	154	1.28	0.0478
Epithelium development	6	1069	0.74	0.0481
Positive regulation of cellular biosynthetic process	8	2041	0.59	0.0494

FDR:false discovery rate

MF

term description	observed	backgrour	strength	FDR
Molecular function regulator activity	12	1960	0.78	0.00028
Enzyme regulator activity	9	1239	0.85	0.0035

CC

term description	observed	backgrour	strength	FDR
Cyclin-dependent protein kinase holoenzyme comple	3	51	1.76	0.0438

KEGG

term description	observed	backgrour	strength	FDR
Progesterone-mediated oocyte maturation	5	95	1.71	1.49E-05
Cellular senescence	5	150	1.52	5.85E-05
Breast cancer	5	146	1.53	5.85E-05
Melanoma	4	72	1.74	7.91E-05
EGFR tyrosine kinase inhibitor resistance	4	77	1.71	8.18E-05
Cell cycle	4	120	1.52	0.00037
FoxO signaling pathway	4	126	1.5	0.00039
Signaling pathways regulating pluripotency of ste	4	141	1.45	0.00052
Gastric cancer	4	146	1.43	0.00053
PI3K-Akt signaling pathway	5	349	1.15	0.00064
Hepatitis B	4	158	1.4	0.00064
MicroRNAs in cancer	4	159	1.39	0.00064
Endometrial cancer	3	58	1.71	0.0008
Rap1 signaling pathway	4	201	1.29	0.001
Proteoglycans in cancer	4	194	1.31	0.001
Pancreatic cancer	3	71	1.62	0.001
Glioma	3	71	1.62	0.001
Acute myeloid leukemia	3	67	1.64	0.001
Non-small cell lung cancer	3	68	1.64	0.001
Ras signaling pathway	4	225	1.24	0.0012
ErbB signaling pathway	3	81	1.56	0.0013
Colorectal cancer	3	82	1.56	0.0013
PD-L1 expression and PD-1 checkpoint pathway in c	3	87	1.53	0.0014
Pathways in cancer	5	515	0.98	0.0018
Prostate cancer	3	97	1.48	0.0018
Choline metabolism in cancer	3	95	1.49	0.0018
HIF-1 signaling pathway	3	102	1.46	0.0019
MAPK signaling pathway	4	286	1.14	0.0022
Human papillomavirus infection	4	324	1.08	0.0034
Phospholipase D signaling pathway	3	147	1.3	0.005
Hepatitis C	3	157	1.27	0.0059
Bladder cancer	2	40	1.69	0.0086
Kaposi sarcoma-associated herpesvirus infection	3	187	1.2	0.0091
Epstein-Barr virus infection	3	192	1.19	0.0095
Human immunodeficiency virus 1 infection	3	203	1.16	0.0108
Regulation of actin cytoskeleton	3	209	1.15	0.0114
Human T-cell leukemia virus 1 infection	3	210	1.15	0.0114
VEGF signaling pathway	2	56	1.55	0.0138
Longevity regulating pathway - multiple species	2	61	1.51	0.0158
GnRH secretion	2	63	1.5	0.0164
Fc epsilon RI signaling pathway	2	65	1.48	0.017
Renal cell carcinoma	2	65	1.48	0.017
Prolactin signaling pathway	2	68	1.46	0.0177
Central carbon metabolism in cancer	2	68	1.46	0.0177
Platinum drug resistance	2	70	1.45	0.0179
Chronic myeloid leukemia	2	75	1.42	0.02
B cell receptor signaling pathway	2	78	1.4	0.0211

Longevity regulating pathway	2	87	1.35	0.0254
Gap junction	2	87	1.35	0.0254
Endocrine resistance	2	94	1.32	0.0283
AGE-RAGE signaling pathway in diabetic complicati	2	96	1.31	0.0289
C-type lectin receptor signaling pathway	2	101	1.29	0.0306
T cell receptor signaling pathway	2	100	1.29	0.0306
Alzheimer disease	3	354	0.92	0.0332
Cholinergic synapse	2	109	1.26	0.0341
Neurotrophin signaling pathway	2	112	1.25	0.0353
Sphingolipid signaling pathway	2	116	1.23	0.0371
Growth hormone synthesis, secretion and action	2	117	1.23	0.0371
AMPK signaling pathway	2	120	1.22	0.0382
Thyroid hormone signaling pathway	2	120	1.22	0.0382
Relaxin signaling pathway	2	126	1.19	0.0405
Autophagy - animal	2	131	1.18	0.0429
Apoptosis	2	131	1.18	0.0429
Apelin signaling pathway	2	133	1.17	0.0429
Insulin signaling pathway	2	132	1.17	0.0429
Estrogen signaling pathway	2	133	1.17	0.0429
