# Supplementary material for: The Potential Impact of HNRNPA2B1 on Human Cancers Prognosis and Immune Microenvironment
Source: J Immunol Res. 2024 Sep 5;2024:5515307. doi: 10.1155/2024/5515307 (PMC11392580; doi:10.1155/2024/5515307)
Supplement: Supplementary 5 — Table 4: relationship between HNRNPA2B1 expression and PFI of each cancer. [file 5515307.f5.pdf]

BP

term description	observed	backgrour	strength	FDR
Positive regulation of cytoplasmic translation	3	15	2.39	0.0056
Regulation of nuclear-transcribed mRNA cataboli	3	26	2.15	0.0124
Regulation of mRNA metabolic process	5	302	1.31	0.0132
miRNA transport	2	3	2.91	0.0193
Nucleobase-containing compound metabolic proces	10	2722	0.66	0.0225
Negative regulation of macromolecule biosynthet	8	1532	0.81	0.0225
Negative regulation of translation	4	169	1.46	0.0225
Negative regulation of cellular biosynthetic pr	8	1592	0.79	0.0225
Positive regulation of nucleobase-containing co	9	2056	0.73	0.0225
Regulation of translation	5	456	1.13	0.0237
Macromolecule localization	9	2356	0.67	0.0237
Negative regulation of cellular process	12	4736	0.49	0.0237
Response to chemical	11	4010	0.53	0.0312
Negative regulation of gene expression	6	899	0.91	0.0342
CRD-mediated mRNA stabilization	2	11	2.35	0.0342
Positive regulation of macromolecule biosynthet	8	1935	0.71	0.0348
Cellular response to chemical stimulus	9	2609	0.63	0.0348
Negative regulation of nuclear-transcribed mRNA	2	12	2.31	0.0348
Negative regulation of mRNA metabolic process	3	92	1.6	0.0348
Cellular localization	9	2677	0.62	0.0385
Radial glial cell differentiation	2	14	2.25	0.0385
Regulation of catabolic process	6	988	0.87	0.0393
Positive regulation of cellular biosynthetic pr	8	2041	0.68	0.0393
Positive regulation of macromolecule metabolic	10	3533	0.54	0.0407
mRNA metabolic process	5	611	1	0.0407

FDR: false discovery rate

MF

term description	observed	backgrour	strength	FDR
miRNA binding	4	33	2.17	9.27E-05
mRNA binding	6	326	1.36	0.00025
RNA binding	9	1672	0.82	0.0019
Organic cyclic compound binding	14	6050	0.45	0.0034
Heterocyclic compound binding	14	5977	0.46	0.0034
Single-stranded RNA binding	3	86	1.63	0.0286
Protein binding	14	7242	0.38	0.0286
G-rich strand telomeric DNA binding	2	10	2.39	0.0286
Gamma-catenin binding	2	13	2.28	0.0289
Identical protein binding	8	2144	0.66	0.0433
Single-stranded DNA binding	3	120	1.49	0.0441

CC

term description	observed	backgrour	strength	FDR
Extracellular exosome	12	2096	0.85	5.35E-06
Extracellular space	13	3247	0.69	9.44E-06
GAIT complex	2	4	2.79	0.0024
Ribonucleoprotein complex	6	687	1.03	0.0025
Spliceosomal complex	4	197	1.4	0.0035
CRD-mediated mRNA stability complex	2	6	2.61	0.0035
Histone pre-mRNA 3end processing complex	2	6	2.61	0.0035
Endomembrane system	12	4721	0.5	0.0038
Catalytic step 2 spliceosome	3	90	1.61	0.008
Integral component of presynaptic active zone m	2	16	2.19	0.0128
Protein-containing complex	12	5506	0.43	0.0162

Catenin complex	2	31	1.9	0.0365
Cell periphery	12	6015	0.39	0.0365
Cytoplasm	16	12056	0.21	0.0379
Intracellular membrane-bounded organelle	16	12149	0.21	0.0409
